# Geochemical Transformations of Gypsum Under Multiple Environmental Settings and Implications for Ca-Sulfate Detection on Mars

**DOI:** 10.1021/acsearthspacechem.4c00137

**Published:** 2025-02-28

**Authors:** Merve Yeşilbaş, Tuan H. Vu, Robert Hodyss, Olivier Poch, Bernard Schmitt, Mathieu Choukroun, Paul V. Johnson, Janice L. Bishop

**Affiliations:** † Carl Sagan Center, SETI Institute, Mountain View, California 94043, United States; ‡ Department of Chemistry, Umeå University, Umeå SE-90187, Sweden; § Jet Propulsion Laboratory, California Institute of Technology, 4800 Oak Grove Drive, Pasadena, California 91109, United States; ∥ Univ. Grenoble Alpes, CNRS, IPAG, Grenoble 38000, France; ⊥ NASA Ames Research Center, Moffett Field, California 94035, United States

**Keywords:** gypsum, bassanite, anhydrite, vibrational spectroscopy, XRD, thermal dehydration, sulfate-Cl salts interactions

## Abstract

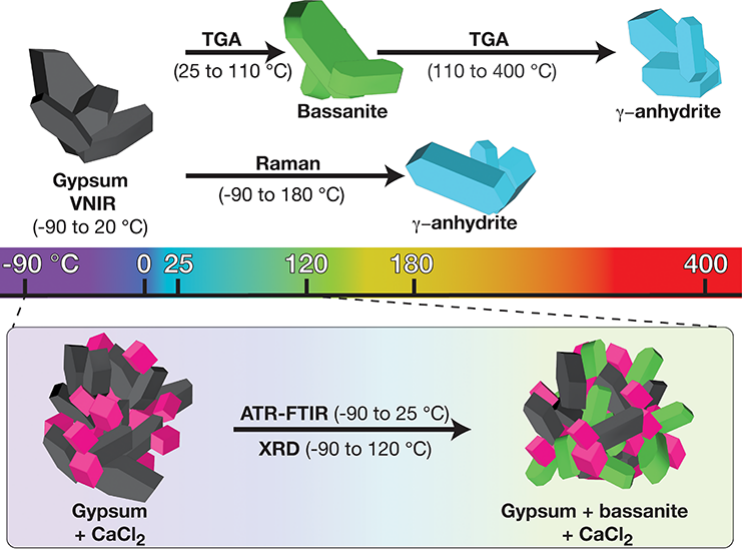

Calcium sulfate minerals are found in multiple environments on Earth and Mars, with chloride (Cl) salts widely distributed on both planets. Low-temperature studies have explored geochemical processes, including the formation of transient liquid water and ion migration on Mars. Some Cl-salts (e.g., NaCl and CaCl_2_) can dissolve gypsum (CaSO_4_·2H_2_O) in certain environments, making gypsum-Cl salt interactions significant. Additionally, gypsum’s geochemical transformation at high temperatures reveals dehydration pathways crucial for understanding Mars’ aqueous history and potential for life. This study examines gypsum dehydration through (i) thermal analyses and (ii) interactions with Cl-salts over a temperature range of −90 to 400 °C. We applied three spectroscopic techniques (Raman, visible/near-infrared, and mid-IR) plus X-ray diffraction (XRD) to analyze these samples under variable conditions. This study also provides a low-temperature spectral data set for gypsum and gypsum-Cl salt mixtures, beneficial for orbital analyses. Our findings reveal that experimental (i) heating rates, (ii) temperature ranges, (iii) relative masses of gypsum and Cl-salts, and (iv) dehydration environments (e.g., in situ and in vacuo) influence Ca-sulfate phase formation. Although we find different results in some cases, this study demonstrates that changing experimental conditions affects the detectability and transformation of gypsum. Further, these results indicate that the geochemical environmental conditions on Mars play a role in gypsum’s geochemical transformation to dehydrated components. This study also provides structural and chemical data for Ca sulfate assemblages from vibrational spectroscopy and XRD, which extends our knowledge of gypsum and related materials under variable conditions, thus aiding orbital and surface planetary analyses that may help to advance our understanding of planetary geochemistry on Mars.

## Introduction

Ca-sulfate hydrates, gypsum (CaSO_4_·2H_2_O), and bassanite (CaSO_4_·0.5H_2_O; hemihydrate) are of great interest to the scientific community due to their significance in terrestrial and planetary environments^1−5^ as well as in technological applications.^6−9^ Anhydrite CaSO_4_, their dehydrated counterpart, forms three distinct phases (α-, β-, and γ-CaSO_4_) of contrasting crystal structure and thermal stability.^10^ In particular, the γ-anhydrite phase, also known as soluble anhydrite, is found in terrestrial settings.^11^ It can be a metastable phase that occurs when gypsum or bassanite dehydrates at high temperatures (110–300 °C).^12^ Gypsum is commonly found in many terrestrial settings on Earth, such as in marine evaporative environments and hydrothermal veins.^5,13^ In addition, gypsum was detected in several locations on Mars via orbital analyses. For example, the *Observatoire pour la Minéralogie, l’Eau, les Glaces et l’Activité* (OMEGA) detected gypsum at Mars’ North Pole in Olympia Undae.^14,15^ Gypsum was also detected at Meridiani Planum using Compact Reconnaissance Imaging Spectrometer for Mars (CRISM) spectral imaging^16^ and on the surface using thermal emission spectroscopic analyses by the Opportunity rover.^17^ The *Curiosity rover* detected gypsum together with bassanite and anhydrite^4,18^ in Gale Crater via its Chemistry and Mineralogy (CheMin) instrument.^19^ Bassanite has also been detected at Mawrth Vallis by CRISM.^20^

Gypsum and anhydrite have been found together in many marine evaporative deposits on Earth, but bassanite is a metastable phase that occurs only when the relative humidity, temperature, and pH conditions are such that gypsum and anhydrite phases are in transition. Anhydrite formation occurs at higher temperatures than gypsum formation and at lower temperatures, salt concentrations, and pH than bassanite formation.^5^ Gypsum can lose its structural water through subhemihydrates that form the more stable phases bassanite and anhydrite. This transformation happens when temperatures are above ∼95–100 °C^5,8,12^ or salts are present at ∼90 °C.^5^ Dehydrated bassanite and gypsum forms can coexist during these processes, and previous studies reveal that applied heating rate, temperature, and the salts’ solubility promote the dehydration of gypsum.^2,6,13^ The dehydration pathway of gypsum and its detection are still enigmatic, but literature studies suggest two ways gypsum can transform: (i) gypsum to bassanite and then anhydrite from gypsum powders or (ii) gypsum directly transforms into the anhydrite from larger particles of gypsum crystals.^21−23^

Previous studies highlight that humidity and temperature affect the geochemical transformation of gypsum, bassanite, and soluble anhydrite.^2,5−8,24^ For example, laboratory studies show that soluble anhydrite can readily transform to bassanite, even under low humidity.^2,5^ This was also observed in several places on Earth, where the bassanite found in the marine evaporative environments also transforms back to gypsum in high humidity environments and during diurnal cycles.^21,25^ Therefore, it is highly likely that these geochemical transformations also occur on Mars, as its environmental conditions support low water/rock ratios and low temperatures, which allow for the existence of all Ca-sulfate phases.^4,26^ Therefore, the accurate detection of these phases, especially from orbital analyses, is important for understanding the aqueous geochemical history of Mars.

The in situ and orbital analyses on Mars use different types of instruments with different detection limits. These consequently provide challenges for identifying and characterizing Ca-sulfate phases of different hydration states. For example, the CheMin X-ray diffraction (XRD) instrument’s angular resolution is 2θ ∼ 0.3°, which makes identification of bassanite and soluble anhydrite phases below 20° (2θ) difficult, as this resolution provides no distinctive reflections for these phases.^4^ We should also note that the XRD analyses of soluble anhydrite and bassanite, even with a high-resolution instrument under controlled laboratory conditions, reveal challenges due to their significant similarities in crystal structure.^2,11^ However, the SuperCam instrument on the *Perseverance* rover is equipped with a green, 532 nm, pulsed laser, is one of the first planetary Raman spectrometers, and also has a visible/near-infrared (VNIR) reflectance spectrometer (400–900 nm, 1.3–2.6 μm). SuperCam can be employed at a distance of 7 m from the focal point, and its optical design enables the detection of minerals and organic materials (e.g., carbonates and sulfates), which are strong Raman scatterers.^27^ This also shows the importance of collecting laboratory Raman spectra using a green laser and VNIR reflectance spectra, especially at low temperatures, to support these SuperCam analyses. Further, orbital analyses using data acquired by the CRISM^28^ and OMEGA^29^ imaging spectrometers have enabled identification of hydrated Ca-sulfate phases, including gypsum^30−32^ and bassanite,^20,33^ as well as a wide variety of mono- and polyhydrated sulfates^34^ detectable in the VNIR region. The shape and position of the hydration bands near 1.4 to 1.5, 1.9 to 2.0, and 2.4 μm in the NIR region are used to identify varied Ca-sulfate phases.^5,13,35,36^ As anhydrite has no features in these spectral ranges, it is more challenging to identify anhydrite via CRISM and OMEGA images.^5^ Furthermore, the upcoming ExoMars rover mission, now expected to launch in the 2030s, will deploy several spectrometers, including a VNIR instrument (e.g., Mars MultiSpectral Imager for Subsurface Studies; Ma_MISS (0.4–2.2 μm)),^37^ a visible plus IR spectrometer (e.g., MicrOmega),^38^ and the RLS Raman spectrometer,^39^ that will together allow for further probing and identification of minerals and organics on Mars. The IR and Raman measurements will also be used to identify all mineral phases, including crystalline and amorphous minerals, inside the Martian regoliths.^39^

The development of these instruments and demand for space missions highlight the need for more laboratory experiments to evaluate spectra collected from orbit and on the surface. In particular, low-temperature vibrational and XRD spectra of the Ca-sulfate minerals are essential for accurate identification of these minerals as measured at Mars. Many literature studies have analyzed the dehydration and transformation of Ca-sulfate minerals using varied experimental and modeling techniques,^2,6,7,10,12,23,40,41^ but only a few studies have highlighted the transformation and solubility of Ca-sulfate phases in the presence of other salts at low temperatures.^13,41−46^ To fill this knowledge gap, this work provides a comprehensive study on the dehydration pathways of gypsum and gypsum-Cl salt mixtures in an extended temperature range of −90 to 400 °C with commonly used techniques (VNIR reflectance, mid-IR, Raman, XRD, and thermal decomposition) by the research community.

## Methods

### Gypsum Source and Characterization

The gypsum (JB1464) used in this study was collected from Niedersachswerfen (Nordhausen, Harz, Germany) and purchased from the Gunnar Farber Mineralien Company. The gypsum rock was crushed using a mortar and pestle and then dry sieved <45 μm. After sieving, the morphology and grain size distribution of the gypsum were characterized using scanning electron microscopy (SEM; Zeiss Electron Microscopy) (Figure S1a). Then, the elemental composition of gypsum was measured using energy dispersive spectroscopy (EDS). We analyzed 3 images and chose 4 particles for each image to determine the average particle size distribution, which varied from very fine particles to larger crystals ranging from 10 to 250 μm. Further elemental composition analyses by EDS also confirmed that the atomic weight percentage of the gypsum grains was mainly composed of calcium sulfate with a small amount of phosphorus (Figure S1b).

### Cryogenic Experiments

For cryogenic experiments, we used several techniques and different gypsum-Cl salt (NaCl and CaCl_2_) ratios to explore the spectral features of gypsum in varied salty environments on Mars. Depending on the techniques, we used temperature ranges from −90 to 25 °C or from −90 to 180 °C with varied heating rates. To summarize our experiments, we present the experimental parameters (e.g., samples, temperature range, heating rate, and dehydration environments) in Table 1.

**Table 1:** Summary of the Experimental Parameters in This Study

experimental techniques	sample	temperature range (°C)	heating rate (°C/min)	dehydration environment
TGA (Umeå University)	gypsum powder	25 to 400 °C	1	N_2_ (g)
TPD-FTIR (Umeå University)	gypsum powder	25 to 400 °C	10	in vacuum
cryogenic-FTIR (Umeå University)	gypsum paste (50 g/L)	–90 to 25 °C	10	N_2_ (g)
	gypsum-CaCl_2_ (50 g/L)	–90 to 25 °C	10	N_2_ (g)
	gypsum-NaCl (50 g/L)	–90 to 25 °C	10	N_2_ (g)
cryogenic-VNIR (IPAG-CSS)	gypsum powder	20 to −90 °C	6	in vacuum
cryogenic-VNIR (JPL)	gypsum paste (50 g/L)	–90 to 25 °C	10	in vacuum
	gypsum-CaCl_2_ (50 g/L)	–90 to 25 °C	10	in vacuum
	gypsum-NaCl (25 g/L)	–90 to 25 °C	10	in vacuum
cryogenic-Raman (JPL)	gypsum paste (50 g/L)	–90 to 180 °C	30	N_2_ (g)
	gypsum-CaCl_2_ (50 g/L)	–90 to 120 °C	30	N_2_ (g)
	gypsum-NaCl (25 g/L)	–90 to 150 °C	30	N_2_ (g)
cryogenic-XRD (JPL)	gypsum paste (50 g/L)	–90 to 120 °C	6	in a closed vessel
	gypsum-CaCl_2_ (50 g/L)	–90 to 120 °C	6	in a closed vessel
	gypsum-NaCl (25 g/L)	–90 to 120 °C	6	in a closed vessel

#### Cryogenic-VNIR

##### Experiments with Gypsum Powder at the University of Grenoble, IPAG, Cold Surface Spectroscopy Laboratory

The 45 μm gypsum powder was placed in a cylindrical aluminum sample holder 14 mm in diameter and 3 mm deep. The surface of the sample was flattened with a spatula. We filled the sample holder with 0.1778 g of gypsum powder, for which the estimated porosity was 83%, and the density was 0.33 g/cm^3^. The reflectance spectra were measured from 0.4 to 2.7 μm using the spectro-gonio radiometer SHINE (SpectropHotometer with variable INcidence and Emergence)^47^ and the environmental simulation chamber CarboN-IR, part of the Cold Surface Spectroscopy Facility (CSS; https://cold-spectro.sshade.eu) at the Institut de Planétologie et d’Astrophysique de Grenoble (IPAG). CarboN-IR is a stainless-steel cylindrical chamber containing a copper inner cell, where the sample holder is placed. Both the main chamber and the inner cell have sapphire windows to allow illumination and observation of the sample from the visible to the mid-infrared. The chambers can be evacuated to a high vacuum using a pumping system. First, the inner cell containing the sample was pumped down to about 10^–6^ mbar for 80 min at 20 °C to desorb some water adsorbed on the sample. Then, a pressure of 11 mbar of pure N_2_ (g) was introduced into the inner cell to ensure good thermalization. The temperature inside the cell is controlled with a helium cryostat, a heater, and a silicon diode temperature sensor connected to a proportional-integral-derivative (PID) temperature controller. The sample was illuminated with a monochromatic beam 7.5 mm in diameter at normal incidence, and the reflected light is observed at an emergence angle of 30° with two visible and near-infrared detectors. The spectral sampling was 20 nm from 0.4 to 1.3 μm, 5 nm from 1.3 to 2.4 μm, and 10 nm from 2.4 to 2.7 μm. The spectral resolutions were 5 nm from 0.40 to 0.66 μm, 10 nm from 0.68 to 1.6 μm, and 20 nm from 1.6 to 2.7 μm. Surfaces of Spectralon and Infragold (LabSphere Inc.) were measured from 0.4 to 1.32 μm and from 1.25 to 2.7 μm, respectively, under the same conditions as the samples and used as references to calibrate the signal measured on the samples and to obtain absolute values of reflectance factors, as described in Potin et al. (2018).^48^

##### Experiments with Gypsum and Gypsum-Salt Pastes at JPL Facilities

To confirm the phase variations from H_2_O/Cl ice (NaCl·2H_2_O and CaCl_2_·6H_2_O) in gypsum, a Thermo Nicolet 6700 Fourier Transform Infrared (FTIR) spectrometer equipped with a Pike Tech DiffusIR reflectance accessory (30° nominal angle of incidence) and a low-temperature vacuum chamber was used. The background spectrum was recorded in rough vacuum (∼5 mTorr) at −90 °C using a 600-grit gold diffuse reflectance standard (ThorLabs) with 2 cm^–1^ spectral resolution.

50 mg of gypsum were mixed with (i) 0.1 mL of distilled water (500 g/L), (ii) 0.1 mL of 1% wt CaCl_2_, and (iii) 0.2 mL of 1% wt NaCl to create a thick paste inside a gold sample cup holder that coupled to the IR reflectance accessory under standard atmospheric pressure conditions (∼754 Torr) at 25 °C. This was followed by the evacuation of the sample chamber to 5 mTorr at 25 °C, and the sample was frozen to −90 °C with a cooling rate of 30 °C/min, where the fast-freezing process from 25 to −90 °C took place in about 4 min. After stabilizing the sample in these environmental conditions, spectra were collected as the sample was heated at a rate of 10 °C/min up to 25 °C. Each spectrum was collected with 1000 coadded scans with a spectral resolution of 2 cm^–1^.

#### Cryogenic-FTIR at Umeå University

To examine the phase variations from H_2_O/Cl ice to H_2_O bound in gypsum as crystalline water, we used FTIR spectroscopy coupled with the attenuated total reflectance (ATR) thermal stage for cryogenic measurements (single bound diamond by Golden Gate, serial number N29328 by Specac). We mixed 50 mg of gypsum samples with 0.1 mL of distilled water or 0.1 mL of 1% wt NaCl or 1% wt CaCl_2_ solutions and let them equilibrate for 15 min. A 10 μL aliquot of the aqueous suspensions was transferred on the ATR stage at 25 °C and then rapidly frozen to −90 °C over 5 min. Next, we ensured that the sample was frozen by sequentially collecting spectra until the intensities of the dominant bands of water were constant for 10 min. Next, the samples were heated at a rate of 10 °C/min from −90 to 25 °C.

All spectra were measured using a Bruker Vertex 70/V FTIR spectrometer equipped with a DLaTGS detector. We probed the spectra in H–O–H bending and SO_4_ stretching regions (1800–1000 cm^–1^) with a 4 cm^–1^ spectral resolution, adding 100 scans per spectrum every 89 s at a 10 Hz forward/reverse scanning rate. These collected spectra were treated with the Blackman-Harris three-term apodization function with a 16 cm^–1^ phase resolution and Mertz phase correction algorithm.

#### Cryogenic-Raman at JPL Facilities

The Raman spectra were collected with a Horiba Jobin-Yvon (LabRam HR) dispersive confocal Raman microscope equipped with a liquid-nitrogen-cooled cryostage (Linkam LTS 350), which helped us perform the experiments in situ and over the desired temperature range between −90 and 180 °C. A frequency-doubled Nd/YAG green laser (532 nm, 50 mW) was used as the excitation source for the Raman spectra, and a 50× Olympus BXFM confocal microscope with a 50× lens was used to focus the laser beam and collect the signal from the frozen samples. The spectral calibration was performed at 25 °C, using the sharp feature of a Si wafer at 520.7 cm^–1^. Spectra were collected with a 600 grooves/mm grating filter at 1.5 cm^–1^ spectral resolution.

Raman spectra were acquired over the 100–4000 cm^–1^ spectral range from −90 to 180 °C to reveal dehydration of gypsum and probe if any other Ca-sulfate phases formed. The same sample preparation procedure was used as the cryogenic VNIR measurements at JPL. Prepared samples were inserted into the cryostage at 25 °C and then flash-frozen to −90 °C with a cooling rate of 30 °C/min. After the frozen paste was equilibrated at −90 °C for 15 min, the temperature was increased to 180 °C with a heating rate of 30 °C/min.

#### Cryogenic-X Ray Diffraction at JPL Facilities

A portion of each prepared sample, as described for cryogenic VNIR and Raman experiments, was transferred to 0.9 mm diameter borosilicate capillaries, which were then flame-sealed and mounted on the goniometer of a Bruker D8 Discover Da Vinci X-ray Diffractometer at ambient conditions. The samples were then flash-frozen to −90 °C using an Oxford Cryostream 800 precooled at this temperature (precision within ±1 K). After the XRD pattern was acquired at −90 °C, the samples were heated at 6 °C/min until the temperature reached 120 °C. XRD patterns were collected with a 2θ angular resolution of 0.02° using a Cu Kα X-ray source (λ = 1.5406 Å) and a linear energy-dispersive LynxEye XE-T 1D detector.

### Thermal Decomposition at Umeå University

The gypsum mineral was decomposed by thermal gravimetric analysis (TGA) and temperature-programmed desorption (TPD)-FTIR to monitor the loss of structural water in the gypsum mineral. The amount of water loss associated with specific temperatures was extracted by TGA, while spectral changes were monitored by TPD-FTIR.

About 4 mg of gypsum mineral was heated from 25 to 400 °C with a heating rate of 1 °C/min under a N_2_ (g) environment in a Mettler Toledo TGA/DSC-1 machine. TGA experiments were operated in two stages: (i) the mineral powders were heated to 80 °C for 180 min to eliminate weakly adsorbed surface water, and (ii) these mineral powder samples were then heated to 400 °C to identify the loss of remaining structural water.

In TPD-FTIR experiments, the gypsum powder was deposited on a tungsten mesh (Unique Wire Weaving Co.a, Inc., mesh diameter of 0.002 in.) and then pressed with 5 N/m force. Then, the prepared sample was placed between the steel discs and inserted into the copper heating shaft. The sample was dried at 25 °C in vacuo (<0.07 Torr) for 10 min in a transmission optical reaction chamber (AABSPEC #2000-A) equipped with CaF_2_ windows. During this time, FTIR spectra were collected to ensure that the loss of water was stable. The sample was then heated from 25 to 400 °C with a heating rate of 10 °C/min. Spectra were collected using a Bruker Vertex 70/V FTIR equipped with a DLaTGS detector through the spectral range of 1100–4000 cm^–1^ at 4 cm^–1^ spectral resolution. The same spectral correction algorithms were applied, as described for cryogenic-FTIR measurements.

## Results and Discussion

### Spectral and Structural Signatures of Gypsum and Gypsum-Cl Salts’ Dehydration

We investigated whether the presence of Cl-salts affected gypsum dehydration and whether the temperature affected the formation of new dehydrated phases from gypsum. To confirm the presence of any dehydrated phases, we used cryogenic-VNIR experiments to probe the H_2_O and SO_4_ overtones and combination modes of gypsum. The first set of VNIR experiments was performed in vacuo conditions between +20 and −90 °C at the CSS facility. We used a slower cooling rate for these experiments, where the cooling from 20 to −90 °C took 660 min.

**Figure 1:**
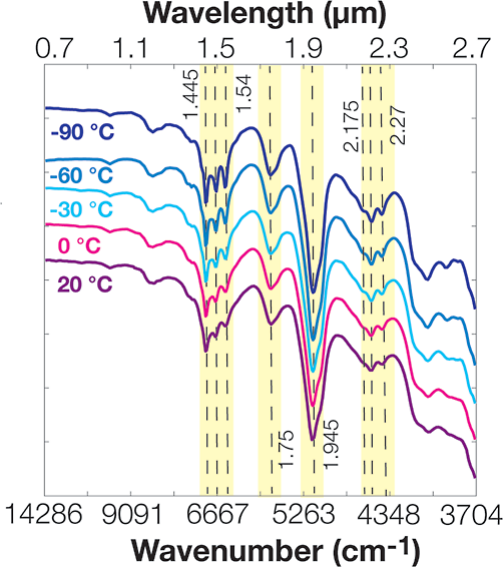
Visible near-IR reflectance spectra of gypsum at low temperature. Freezing of gypsum powder from 20 to −90 °C provided more distinct H_2_O combinations and overtone modes in the 1.445, 1.49, and 1.54 μm triplets and at 1.75 and 1.945 μm, and in the 2.175, 2.22, and 2.27 μm triplet.

Our experiments revealed that freezing gypsum powders from 20 to −90 °C produced sharper spectral bands for the H_2_O combination and overtone modes. Three distinct bands at ∼1.445, 1.49, and 1.54 μm that were attributed to the H_2_O bending and stretching combination modes^5,49^ gave sharper bands upon freezing (Figure 1). The same trend was observed for the other H_2_O and SO_4_ combinations and overtone band features at 1.75 and 1.945 μm as well as for triplet bands at 2.175, 2.22, and 2.27 μm.

**Figure 2:**
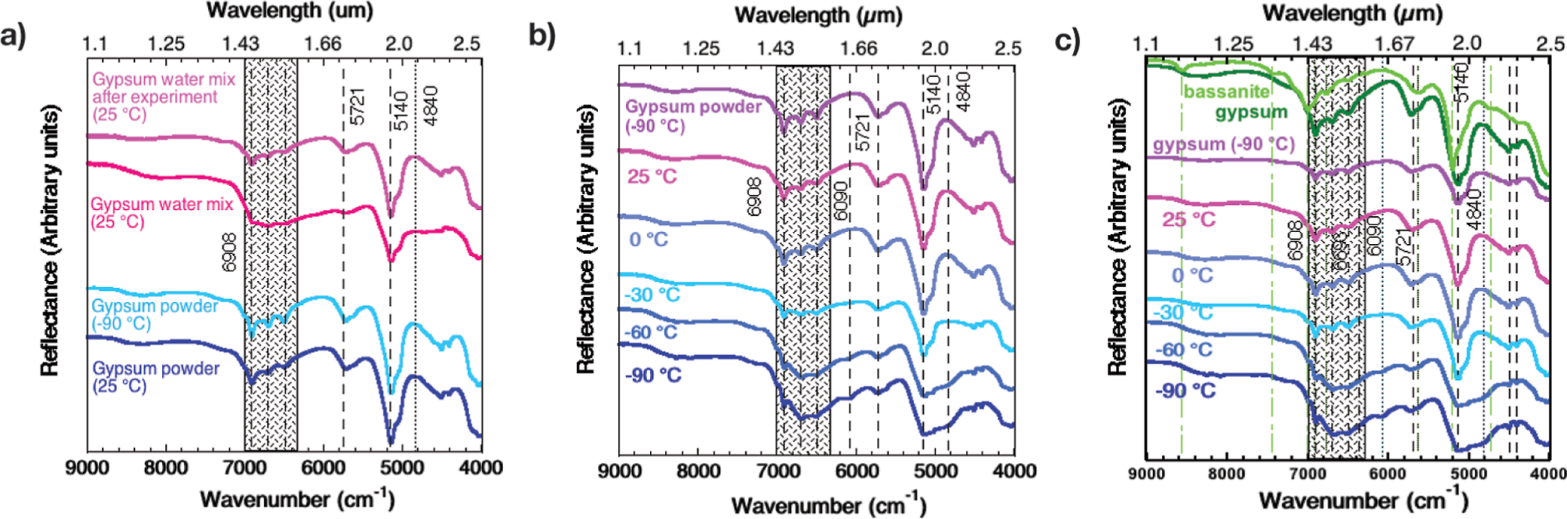
(a) Near-IR reflectance spectra of gypsum and mixtures with salts. Comparison of gypsum powder at 25 °C and −90 °C, where H_2_O bending and stretching combination modes in the 1.44–1.54 μm spectral region became more distinct at −90 °C, which could be related to the temperature effect (bottom). We observed the same effect in gypsum paste (pink) at 25 °C with the spectrum of vacuum-dried gypsum paste evident at the same temperature (purple). Similar behavior when flash-frozen (b) gypsum-NaCl and (c) gypsum-CaCl_2_ pastes were heated from −90 to 25 °C.

For comparison, we investigated the gypsum spectral bands at JPL when the sample was frozen as a hydrated paste and as a powder at −90 °C and then when both samples were heated to 25 °C. The VNIR spectra of gypsum powder at −90 °C present sharper bands of the H_2_O bending and stretching vibrations than those at 25 °C (Figure 2), which confirmed our results from CSS (Figure 1). However, the experiment using a hydrated gypsum paste revealed a broader spectral band in the ∼6090–6910 cm^–1^ (1.447–1.64 μm) spectral region, where the three distinct gypsum spectral features at 6904 (1.448 μm), 6700 (1.492 μm), and 6508 cm^–1^ (1.537 μm) were only barely observed, likely due to the additional water in the sample producing the broad band around 1.5 μm. The presence of ice or frost on gypsum particles may also mask these bands. This characteristic gypsum triplet appeared when the samples were heated to 25 °C (Figure 2a).

Our experiments with gypsum-Cl salt mixtures showed spectral features consistent with water frost together with the gypsum at −90 °C that changed to bands only due to gypsum as the temperature was increased (Figure 2b,c). Spectra of the gypsum/NaCl and gypsum/CaCl_2_ samples followed the same trend as that of gypsum paste. When the Cl-salt mixtures were frozen, H_2_O combination and overtone bands at 1.445, 1.49, and 1.54 μm triplets and 2.175, 2.22, and 2.27 μm triplets were masked by ice and frost. All these characteristic gypsum bands became distinct when the samples were heated to 25 °C. We found that neither gypsum nor gypsum with 1% wt of NaCl pastes at 25 °C presented spectral features like bassanite or anhydrite as reported in previous studies.^3,5^ The spectra of these gypsum/NaCl and gypsum/CaCl_2_ samples exhibited the same spectral features as those of dried gypsum powder at 25 °C (Figure 2c).

**Figure 3:**
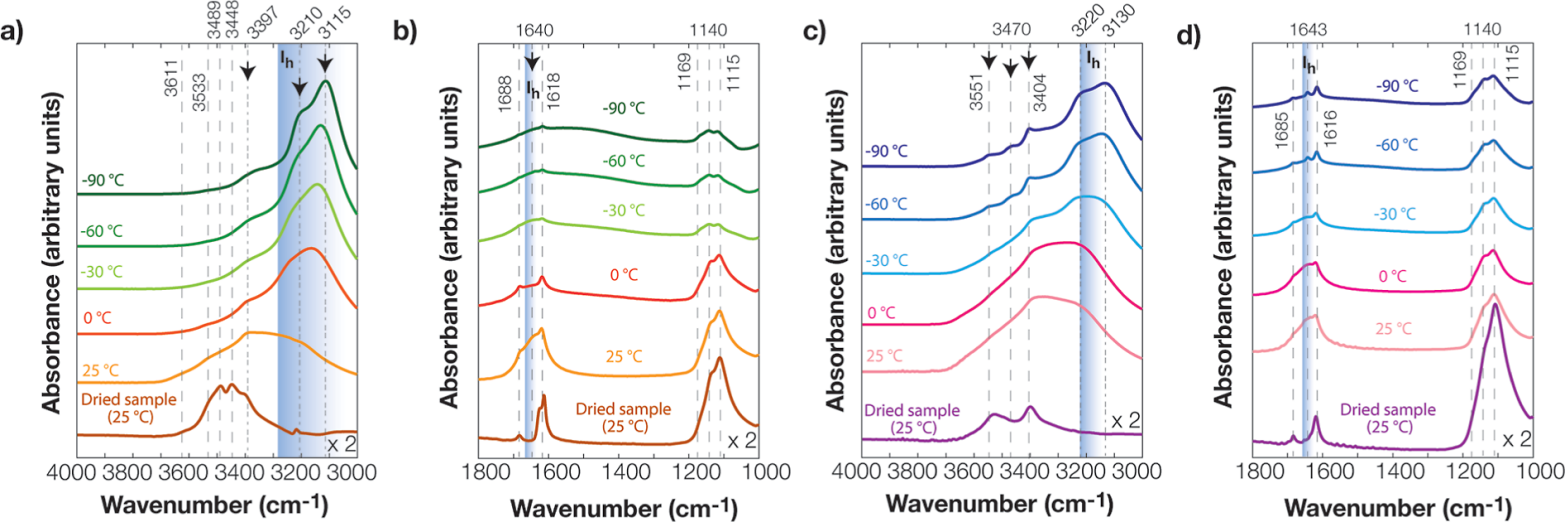
Cryo-FTIR analyses of gypsum-CaCl_2_ paste from flash-frozen paste at −90 °C to dried paste form at 25 °C in (a) H_2_O stretching and (b) H_2_O bending spectral regions. Flash-frozen gypsum-NaCl paste at −90 °C heated to 25 °C and dried in (c) H_2_O stretching and (d) H_2_O bending spectral regions. Down arrows indicate a decrease in the spectral intensity.

In the cryogenic-FTIR experiments, measurements were designed with more hydrated samples (250 g/L) than for VNIR, Raman, and XRD to further investigate the interactions between gypsum and chloride salts by detecting structural water loss and any phase changes in the H_2_O stretching (3000–4000 cm^–1^), H_2_O bending, and SO_4_ stretching (1000–1800 cm^–1^) spectral regions. Our cryogenic-FTIR experiments here with gypsum-Cl salt mixtures revealed that gypsum may dissolve more easily in CaCl_2_ than in NaCl solution. This led to dehydration of gypsum by forming the hemihydrate bassanite (CaSO_4_·0.5H_2_O). This was surprising because the samples were only equilibrated for 15 min at ambient conditions, suggesting dehydration of gypsum occurs rapidly. In our previous study,^13^ we also found that gypsum in low concentrations of CaCl_2_ solutions was more soluble than in low concentrations of NaCl solutions. Figure 3a illustrates that the spectrum of gypsum with 1% wt of CaCl_2_ at −90 °C was broader, more like the spectrum of ice^50^ than the spectrum of 1% wt CaCl_2_ at −90 °C.^51^ This finding suggests that the presence of additional water bound to CaCl_2_ came from gypsum dehydration. However, we should note that the cryosalt signatures of CaCl_2_·6H_2_O (antarcticite) and NaCl·2H_2_O (hydrohalite) as well as gypsum exhibit IR-spectral bands at relatively similar band positions for H_2_O bending and H_2_O stretching regions.^52^ The SO_4_ stretching region (∼1000–1200 cm^–1^) was thus used to assess the presence and coordination of sulfate ions in these mixtures. As H_2_O ice dominated the H_2_O stretching and bending regions in the gypsum-CaCl_2_ spectrum at −90 °C, the gypsum spectral bands were revealed as doublets at 1115 and 1140 cm^–1^ with a shoulder band at 1169 cm^–1^. In a separate experiment, the dried thin film of gypsum at −106 °C was recorded in the SO_4_ stretching region and presented in Figure S2. The gypsum presented the most intense SO_4_ band at ∼1109 cm^–1^ with a shoulder band at ∼1140 cm^–1^. This spectrum actually revealed similarities with the spectrum of the dried gypsum/NaCl sample at 25 °C, as shown in Figure 3d. Advancing that, doublet formation at 1115 and 1140 cm^–1^ at −90 °C could be related to the temperature effect and amount of water during the fast-freezing process. Further heating of these samples to −30 °C exhibited no significant change compared to the spectrum at −90 °C; however, at up to 0 °C, the ice bands were progressively decreased, and doublet bands of gypsum and H_2_O-coordinated CaCl_2_ appeared at 1618 and 1688 cm^–1^ (Figure 3b) and ∼3448, 3489, and 3533 cm^–1^ (Figure 3a). This confirmed that in the gypsum-1 wt % CaCl_2_ mixtures, none of these salts, especially CaCl_2_, influence melting the ice below its eutectic point (∼−49 °C), as described in previous studies.^13,53^

We also note that H_2_O ice features were observed up to 0 °C, correlating with the solubility diagram of the gypsum-CaCl_2_ mixtures at low temperature as presented in the FREZCHEM model in a previous study.^13^ Further heating of this sample to 25 °C revealed liquid-like SO_4_–CaCl_2_ bands, including a shoulder ∼3611 cm^–1^ band. After drying the sample with N_2_ (g) at 25 °C for around 20 min, the spectrum exhibited a gypsum-CaCl_2_ mixture similar to a spectrum of volcanic soil mixed with 40% wt CaCl_2_, which corresponds to the spectral signatures of both H_2_O stretch and H_2_O bending regions at ∼3225, 3430, and 3490 cm^–1^ and 1615, 1628, and 1660 cm^–1^.^13^ This confirmed a type of ion-specific interaction took place and the dissolution of gypsum in water.

However, the spectrum of gypsum mixed with 1% wt NaCl at −90 °C was not similar to the spectrum of ice. At–90 °C, ice, gypsum, and even hydrohalite signatures revealed in the spectra were similar to those observed in a previous study.^52^ Hydrohalite spectra at −10 °C have spectral bands of ∼3408, 3462, 3555, 1614, and 1637 cm^–1^, while gypsum spectra at −10 °C (Figure S2) have spectral bands of ∼3403, 3536, 1618, and 1684 cm^–1^. These are relatively close band positions, but the presence of the ∼3462–3470 cm^–1^ band and SO_4_ region spectral bands were ∼1115, 1140, and 1169 cm^–1^ and confirmed the presence of gypsum and hydrohalite together (Figure 3c,d).^41^ The ice bands (*I*_h_) started to diminish at ∼−30 °C, and these bands were progressively decreased at 0 °C. This resulted in a broader band shifted to higher ∼3400 cm^–1^, caused by the disruption of the H-bonding network of ice, as it became more liquid-like.^50^ The spectrum at 25 °C exhibited a decrease in the intensity of the 3220 cm^–1^ band, revealing the same spectral features at 0 °C. After drying the sample at 25 °C under N_2_ (g) for around 15 min, the gypsum spectrum was observed. This confirmed that 1% wt of NaCl did not dissolve the gypsum as in the case of 1% wt CaCl_2_. This could be related to the ion-specific effects between Cl- and SO_4_-salts, and further investigations with varied types of Cl-salts would be tested for gypsum dehydration. These FTIR experiments were performed under ambient pressure and cryogenic conditions at temperatures of up to 25 °C on both gypsum-CaCl_2_ and gypsum-NaCl samples. These samples revealed liquid-like features by exhibiting a broader spectrum with a major spectral band at ∼3400 cm^–1^. The presence of liquid water could provide an environment for ion-specific reactions of gypsum and Cl salt ions. Drying these gypsum/salt mixture samples at 25 °C confirmed that the CaCl_2_ salt impacts the gypsum dehydration, as presented in Figure 3a,b.

**Figure 4:**
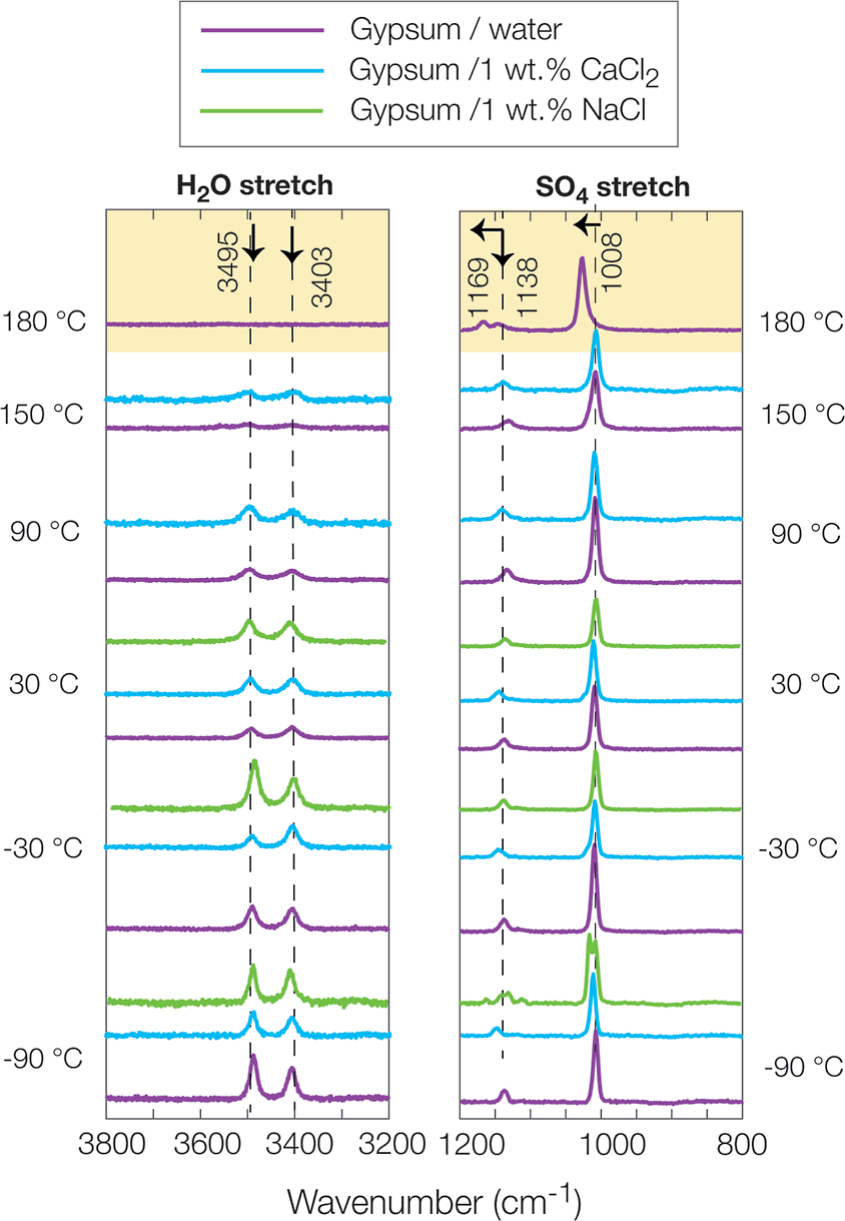
Cryogenic-Raman spectra of gypsum and gypsum-Cl salt mixtures recorded upon heating from −90 to 180 °C. Gypsum paste formed a type of anhydrite, soluble anhydrite, at 180 °C.

Cryogenic Raman experiments provided additional information about the gypsum dehydration in the H_2_O stretching and bending spectral regions as well as the SO_4_ stretching spectral regions (Figure 4). The Raman spectrum of gypsum reveals peaks at the SO_4_ stretching symmetric (ν_1_) and antisymmetric (ν_3_) modes at 1008 and 1138 cm^–1^, respectively, and H_2_O stretching asymmetric and symmetric vibrations^3^ at 3408 and 3496 cm^–1^, respectively. H_2_O stretching bands at cold temperatures are slightly shifted in opposite directions and are sharper than at 25 °C, likely due to the reduced thermal disorder at low temperatures.^50^ The gypsum-NaCl mixture spectrum at −90 °C revealed doublet SO_4_ stretching bands and an additional quadruplet of bands in the ∼1100–1170 cm^–1^ spectral region. These spectral features are more likely due to glauberite, Na_2_Ca(SO_4_)_2_ with its main peak at 1001 cm^–1^ and a quadruplet of bands at 1105, 1140, 1155, and 1170 cm^–1^.^54,55^ However, this spectrum at −90 °C may be related to the deposition of the particles on the cold stage during the fast-freezing process. When heated to −60 °C, these spectral features were lost, and the spectrum displayed only the gypsum features again. After heating from 25 to 150 °C, these H_2_O stretching bands were progressively eliminated until 180 °C. Between 150 and 180 °C, both H_2_O bands were completely lost, similar to the anhydrite spectrum^40^ in the H_2_O stretching region (3000–3800 cm^–1^). This was followed by a corresponding shift in the symmetric SO_4_ tetrahedral vibration (ν_1_) at 1025 cm^–1^ and the splitting of the asymmetric stretch (ν_3_) in a doublet at 1146 and 1169 cm^–1^ at 180 °C (Figure 4). This spectrum shares strong similarities with the γ-anhydrite (soluble anhydrite) spectrum formed through microwave heating at 25 °C^10^ and other Raman spectra of soluble anhydrite.^12,22^ A full spectral data set of the cryogenic-Raman spectra, including all temperature ranges, are presented in Figure S3. Following the gypsum-hemihydrate and anhydrite pathways, Ostroff^45^ found that gypsum from pure Ca-sulfate solutions is not transformed into anhydrite at temperatures below ∼97 °C. Another study^46^ found that increasing the pressure of the gypsum-NaCl aqueous salt mixtures promoted the dehydration of gypsum into anhydrite over an extended temperature range and at all pressures (∼100–450 °C; 1 to 1000 bar). The different dehydrated forms of Ca-sulfates, such as bassanite and anhydrite, from salt-free and with salt solutions at varied temperatures were reported using Raman^56^ and other analytical instruments^57^ and references therein.^57^ These studies pointed out several experimental factors that affect and contribute to the formation pathways of gypsum from solution. These factors include the temperature, the roles of Ca-sulfates as catalysts (e.g., particle size, impurities), ionic concentration of the surrounding environment, and the affinity of the reaction to favor gypsum formation. Applying nonconstant temperature cycles like in our experiments also added “time” as an additional factor, which also makes the heating/cooling rate important to facilitate gypsum dehydration. Furthermore, Jones^58^ reported that the ordering of water molecules from the CaSO_4_ solution plays a significant role in the crystallization and stabilization of varied Ca-sulfate phases upon evaporation, a process that can be related to the amount of water. Our Raman spectroscopy results with gypsum-Cl salt pastes showed that salts have no effect on the formation temperature of soluble anhydrite (around 180 °C). In comparison with previous studies, we have two different important critical factors in our experiments. The first factor was that we analyzed the gypsum-Cl salt pastes where gypsum was more dominant than the salty water in our probed systems. The second factor is that the environmental chamber in the sample stage was exposed to a constant flow of dry *N*_2_ (g) during the Raman spectroscopy experiments. This could result in evaporation of water on the gypsum-NaCl and gypsum-CaCl_2_ pastes by quickly leading to efflorescence of the Cl-salt during our experiments, thereby minimizing the role of Cl-salts in gypsum dehydration.

**Figure 5:**
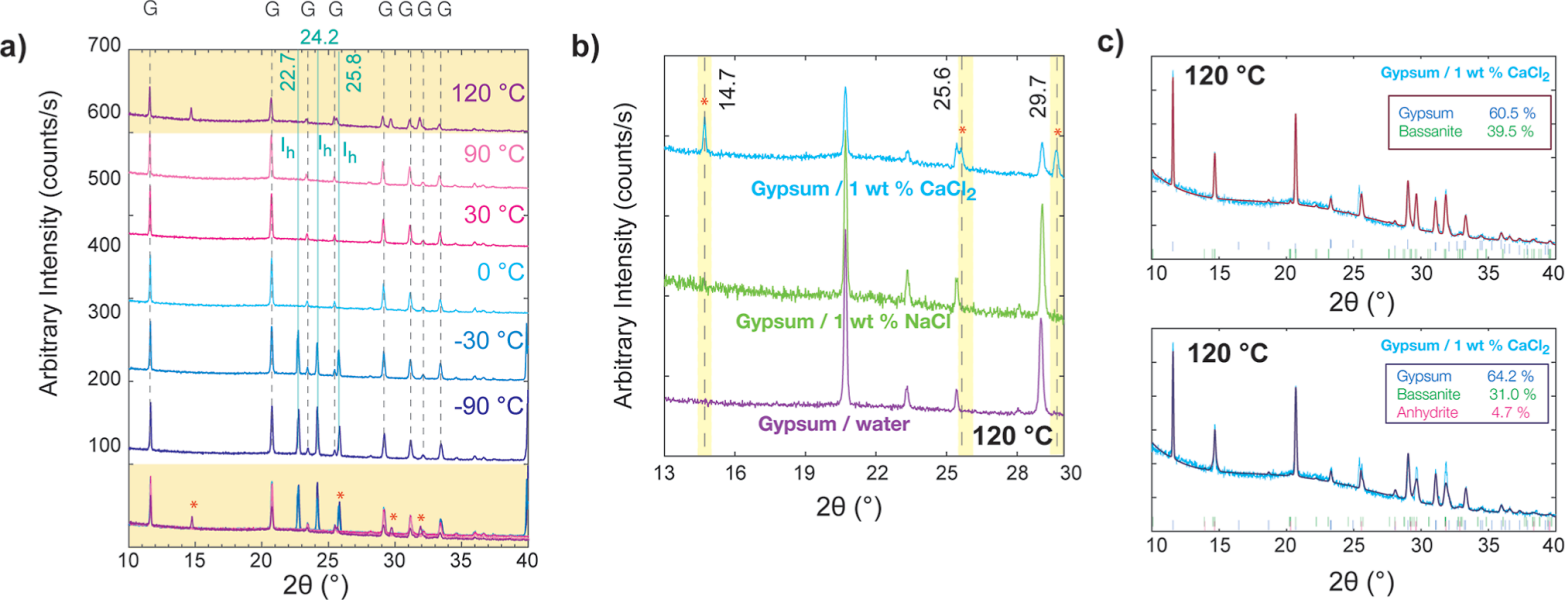
(a) XRD patterns of flash-frozen gypsum-1 wt % CaCl_2_ paste at −90 °C, followed by heating to 120 °C. At this temperature it forms a mixture of gypsum (G) and bassanite and/or soluble anhydrite. Green lines indicate the ice (*I*_h_) XRD reflections at 22.7°, 24.2°, and 25.8°. (b) For comparison, collected XRD patterns of gypsum paste, gypsum-1 wt % NaCl, and gypsum-1 wt % CaCl_2_ pastes at 120 °C are shown. There are similarities between gypsum-1 wt % NaCl and gypsum pastes at 120 °C, whereas gypsum-1 wt % CaCl_2_ paste formed mixtures of gypsum and bassanite or soluble anhydrite. (c) Rietveld refinement analyses for gypsum-bassanite (top, burgundy) and gypsum-bassanite-anhydrite mixture (bottom, navy) were performed to identify the amount and type of gypsum dehydrated phases in gypsum-1 wt % CaCl_2_ paste.

The XRD analysis of dry particulate gypsum exhibits no evidence of ice formation at low temperatures or dehydrated Ca-sulfate phases when heated to 120 °C (Figure S4). However, the gypsum with 1% wt of NaCl and CaCl_2_ pastes presented features of both gypsum XRD patterns (G) and ice (*I*_h_) at ∼22.7°, 24.2°, and 25.8° (2θ),^59^ which correspond to reflections from the 100, 002, and 001 planes, respectively.^60^ These ice bands were eliminated during heating from −90 to 0 °C for gypsum-Cl salt pastes (Figure 5a). When we heated these samples to 120 °C, our experiments revealed that gypsum and gypsum-NaCl pastes exhibit similar XRD patterns (Figure 5b). However, when a mixture of gypsum and 1% wt of CaCl_2_ was heated to 120 °C a new XRD pattern was produced (∼14.7°, 25.6°, 29.7°, and 31.8°), but the intensities of the gypsum peaks (20.7°, 23.4°, 29.1°, and 31.1°) decreased. This could be the signature of a mixture of gypsum and other dehydrated Ca-sulfate phases, reported in previous studies.^2,11,63^ The bassanite and anhydrite phases coexisted during the gypsum dehydration experiments in previous studies.^2,61^ However, the reported temperatures for gypsum dehydration changed, corresponding to the heating rate, the applied temperature, and the environment in which dehydration was carried out.

As shown in Figure 5a,b, gypsum-1 wt % CaCl_2_ XRD patterns revealed additional reflections at 14.7°, 25.6°, 29.7°, and 31.8° 2θ that could be assigned to bassanite or soluble anhydrite.^2,9,61,62^ To resolve coexisting Ca-sulfate phases, we further analyzed the reflections between 28° and 34° (2θ°) as they allow the greatest angular separation between the soluble anhydrite (200), bassanite (220), anhydrite (102), and bassanite (204).^2,9,61,62^ Subsequent Rietveld refinement of the gypsum/CaCl_2_ sample at 120 °C revealed a composition of ∼61–64% gypsum and ∼36–39% additional phases that are consistent with bassanite or a mixture of bassanite/soluble anhydrite (Figure 5c). The XRD data set for the gypsum powder and gypsum-NaCl paste across the full temperature range is presented in Figure S4. Our XRD results confirm that CaCl_2_ can dissolve gypsum at higher temperatures and could result in the formation of partly or fully dehydrated phases. In contrast with our Raman spectroscopy experiments, XRD experiments were performed in a sealed closed vessel that prevents the escape of released water from the gypsum-CaCl_2_ paste. This released water could then lead to the formation of dehydrated phases from gypsum’s reaction with the Cl-salts. Our Raman and XRD results confirm that the presence of water in studied dehydration environments is important to facilitate the dehydration of gypsum. Furthermore, applied heating/cooling rates, the environment in which dehydration takes place, and the molar concentration of Cl-salts are the important factors for gypsum dehydration.

### Structural Water Loss and Thermal Decomposition of Gypsum

**Figure 6:**
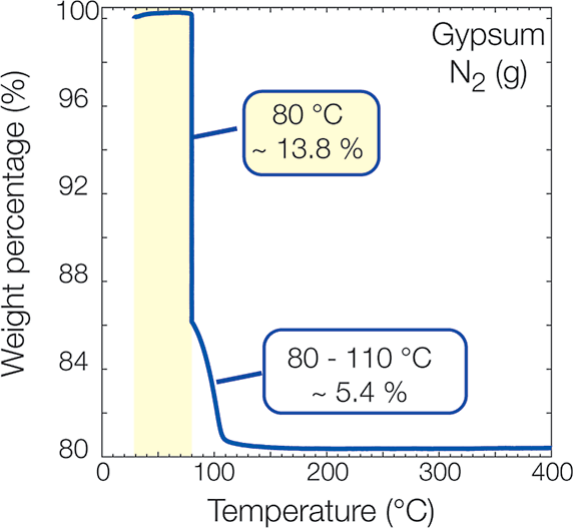
Amount of water loss from gypsum powder, as recorded by TGA under dry N_2_ (g) environment between 25 and 400 °C.

As a first effort to probe the amount of water loss as a function of temperature, TGA analyses were performed. TGA experiments revealed that gypsum lost substantial water (∼13.8% wt) through dehydration at 80 °C. This corresponds to a loss of 1.32H_2_O molecules, so the formed Ca-sulfate phase could be CaSO_4_·1.68H_2_O at 80 °C. This lost water could be related to the surface-adsorbed water and structural water from gypsum. Further loss was obtained by heating from 80 to 110 °C, where gypsum lost ∼5.4% wt of water, and then the water loss remained constant between 110 and 400 °C (Figure 6). The total water loss from gypsum from 25 to 110 °C was 1.84H_2_O molecules, and 0.16H_2_O remained by forming CaSO_4_·0.16H_2_O at 110 °C. Previous studies with gypsum TGA and other thermal analyses (e.g., differential scanning calorimetry and DSC^63^)^6,21,23,40,64^ reported different temperature regimes between ∼100–130 °C (±5 °C) and 180 °C (±10 °C) for the structural water loss from gypsum. These studies reported that gypsum first dehydrates to bassanite between 350 and 450 °C and then to anhydrite and its different forms at higher temperatures between 450 and 1000 °C.^6,10,22^ These temperature differences are related to the dryness of the surrounding gas (e.g., air or N_2_), the heating rate, and experimental procedure.

**Figure 7:**
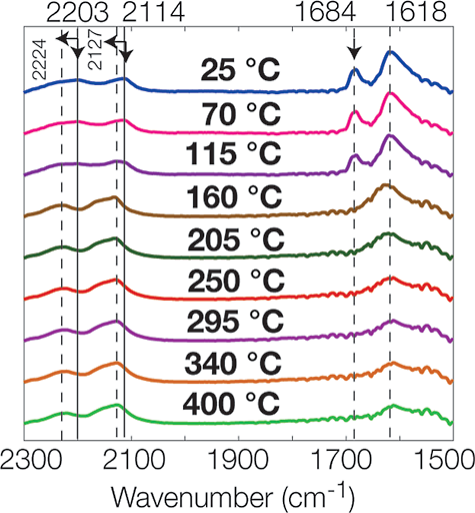
TPD-FTIR analyses of gypsum powder heating from 25 to 400 °C at 10 °C/min in the SO_4_ combination modes in the spectral region (2050–2350 cm^–1^) and the bending mode (ν_2_) of H_2_O.

To reveal what type of Ca-sulfate phase formed during the thermal dehydration process, we conducted temperature-programmed desorption (TPD)-FTIR experiments in the mid-IR region to interpret the structural water loss from gypsum minerals in the H_2_O and SO_4_ bending and combination region, 1500–2300 cm^–1^. Our results revealed a progressive decrease and loss of the 1684 cm^–1^ spectral band of gypsum (ν_2_, H_2_O bending) between 25 and 150 °C (Figure 7). Further heating of gypsum above 160 °C eliminated the 1618 cm^–1^ band (ν_2_, H_2_O bending); however, around 205 °C, this band started to shift to 1613 cm^–1^ at 400 °C. Furthermore, these changes were coordinated in the SO_4_ combination modes spectral region, shifting the 2114 and 2203 cm^–1^ bands to 2127 and 2224 cm^–1^ at 400 °C, respectively.^23^ These results exhibit the weakening of the H-bonding environment could be related to released water molecules in gypsum.^63^ Prasad et al.^23^ probed four spectral bands between 2050 and 2350 cm^–1^, for SO_4_ second-order modes and found them at 2117, 2133, 2200, and 2245 cm^–1^ for gypsum, but our transmission spectra of the gypsum displayed only two broad bands with shoulders. In Prasad et al.^23^ study, FTIR experiments were performed in situ conditions by preparing the gypsum sheet-like samples with different thicknesses (200 and 500 μm) and probing the gypsum single crystals. However, our dehydration experiments were performed under a much drier environment in a vacuum than in the study of Prasad et al.^23^ on bulk gypsum powder. The shift of these two bands around 160 °C was correlated with the complete loss of the lower frequency component (at 2114 cm^–1^ in Prasad et al. 2005^23^) produced by one type of structural water site in gypsum, with a corresponding bending mode at 1684 cm^–1^. The remaining structural water site was depleted progressively at higher temperatures. These results also confirm our TGA results, where we found that the largest amount of water was released at 110 °C, and the release of water remained constant up to 400 °C.

Previous studies^3,5,36,63^ revealed that H_2_O bending modes of Ca-sulfate phases exhibit varied spectral band positions in relation to the type of spectra (e.g., emissivity and absorbance). For example, the H_2_O bending mode of bassanite was found at 1630 cm^–1^ with mid-IR emissivity spectra,^36^ but other studies measuring the absorbance revealed these bands in the ∼1615–1630 cm^–1^ spectral range.^5^ However, some studies have confirmed that there is no H_2_O bending (ν_2_) band for soluble anhydrite.^3^ As our experiments were performed in vacuo with higher temperatures, the formation of bassanite could be expected. Otherwise, a different hemihydrate type of gypsum might be produced for a short time as described by Kyono et al.^65^ such as a hemihydrate intermediate phase containing (0.5 to 0.8) water molecules in the structure. However, we should note that before the formation of bassanite, gypsum, and bassanite could coexist as an intermediate phase and then transform to bassanite at ∼400 °C.

## Applications to Mars

The detection of varied phases of Ca-sulfates is important for understanding the history of water activity, (geo)chemistry, and climate in past environments, including marine and lacustrine systems, both on Earth and Mars. Ca-sulfates could be one of the potential preservers of biosignatures,^11,66^ and this could help us to understand if life has ever existed on Mars. Furthermore, the released water from these sulfates could be used by future human missions on Mars.^41,67^

This work shows that the formation of Ca-sulfate phases probed by the different techniques used herein is affected by (i) experimental heating rates, (ii) temperature ranges, (iii) relative masses of gypsum and Cl-salts in the samples, and (iv) dehydration environments (e.g., in situ and in vacuo). Highlighting this situation is important, as it will raise awareness of potentially conflicting findings that can be made using different techniques, especially when comparing with other modes of measurements (e.g., orbital data). Literature studies^24,68−70^ also pointed out the importance of solubility and water activity terms in CaSO_4_ phase transformations and exploring potential habitability in the (extra)terrestrial environments. In correlation with these studies, we discovered that the presence of water and CaCl_2_ salt (∼1 wt %) dissolves gypsum under ambient conditions.

This work helps for further experimental and modeling studies on the interactions between Ca-sulfates and various Cl salts at low temperatures. It will help us understand how these salts affect gypsum dehydration and link these processes to geochemical activities on Mars and other planetary systems. Additionally, detecting Ca-sulfate and salt interactions at low temperatures using different spectroscopic techniques can enhance data analysis from spacecraft instruments such as MaMISS, MicrOmega, CRISM, RLS, SuperCam, and CheMin.

## Summary and Conclusions

Our experiments have demonstrated two dehydration pathways of gypsum: (i) one where the gypsum loses its structural water and forms dehydrated phases at ∼110 °C and (ii) one where the gypsum interacts with Cl-salts. In the latter, we also observed that samples with high Cl-salt concentrations and the presence of water facilitate the dissolution of gypsum grains. We used thermal dehydration studies and several spectroscopy techniques to examine the dehydration pathways of calcium sulfate hydrates and to detect the resulting phases. Applying controlled (i) heating rates, (ii) temperatures, and (iii) concentrations of Cl-salts as well as the (iv) dehydration environments (e.g., in situ, in a vacuum) influenced gypsum solubility and therefore the formation of dehydrated Ca-sulfates. For example, we prepared the same relative mass of gypsum and Cl-salts for Raman, VNIR, and XRD experiments. The main difference in Raman and XRD experiments was the experimental conditions of the dehydration environments. The released water from gypsum samples was evaporated by dry *N*_2_ (g) during Raman experiments, whereas the XRD experiments were performed in a sealed capillary vessel that limited evaporation of water. Our Raman results revealed that gypsum transformed to soluble anhydrite at 180 °C, but salts had no effect on this temperature. However, XRD experiments from the gypsum-CaCl_2_ sample revealed the formation of gypsum-bassanite/soluble anhydrite mixtures at 120 °C, whereas the gypsum and gypsum-NaCl samples presented no dehydrated phases of Ca sulfate. All these Raman and XRD experimental results revealed that the presence of released water from gypsum samples could affect the formation of other dehydrated phases, and all of these results are related to the dehydration environments, not the experimental techniques. Emphasizing this issue is important to increase awareness of the potentially conflicting findings that can be made using different techniques for gypsum dehydration studies.

VNIR and mid-IR spectroscopy analyses for gypsum revealed distinct H_2_O combinations and overtones of stretching and bending modes that are associated with the dehydration state of gypsum, notably in the VNIR (1.445, 1.49, and 1.54 μm; 2.175, 2.22, and 2.27 μm) and mid-IR (1618 and 1688 cm^–1^; 3402–3539 cm^–1^) ranges. This study uncovered the masking effect of ice and frost on the aforementioned H_2_O combination and bending spectral features of gypsum at low temperatures, and increasing temperatures led to the re-emergence of these bands. The VNIR spectra of gypsum powder at low temperatures also confirmed these results by demonstrating that the temperature had an impact on the H_2_O combination and bending spectral features. Furthermore, VNIR spectra of gypsum-NaCl and gypsum-CaCl_2_ samples exhibited no dehydrated phases of gypsum by heating to 25 °C. However, prepared gypsum and more hydrated gypsum-NaCl and gypsum-CaCl_2_ samples with mid-IR spectroscopy measurements revealed that the gypsum-CaCl_2_ sample formed a mixture of gypsum and bassanite/anhydrite at 25 °C.

Overall, our study indicates that gypsum in an environment containing CaCl_2_ could react under more hydrated environments and higher temperatures to form other less stable Ca-sulfate phases. Furthermore, all these results show the importance of how (i) experimental heating rates, (ii) temperature ranges, (iii) relative masses of gypsum and Cl-salts in the samples, and (iv) dehydration environments (e.g., in situ and in vacuo) all affect the formation of Ca-sulfate phases. We expect this study could provide valuable insights for geochemical processes and improved assessment tools for the characterization of Ca sulfates on Mars and other planetary bodies, including orbital and in situ analyses. While our current findings present some initial pointers to study different dehydration pathways of gypsum, further research is needed to evaluate the other experimental factors to achieve a fundamental understanding. Investigation of the varied dehydrated phases of Ca-sulfate hydrates is vital for assessing the past and present climate and geochemistry of Mars and the other planetary systems where the Ca-sulfate phases are detected through orbital analyses. Additionally, this would be beneficial for advancing our understanding of geochemical systems involving Ca-sulfate phases, such as marine and lacustrine environments in planetary systems.

## Supporting Information Available

SEM-EDS analyses from gypsum, cryogenic ATR-FTIR spectra of flash-frozen gypsum from 25 to −106 °C, cryogenic Raman patterns of gypsum and gypsum-Cl salt mixtures from −90 to 180 °C, and cryogenic XRD spectra of gypsum and gypsum-Cl salt mixtures from −90 to 120 °C (PDF)

## References

[ref1] Wang A.; Zhou Y. Experimental comparison of the pathways and rates of the dehydration of Al-, Fe-, Mg- and Ca-sulfates under Mars relevant conditions. Icarus 2014, 234, 162–173. 10.1016/j.icarus.2014.02.003.

[ref2] Robertson K.; Bish D. Constraints on the distribution of CaSO_4_·*n*H_2_O phases on Mars and implications for their contribution to the hydrological cycle. Icarus 2013, 223 (1), 407–417. 10.1016/j.icarus.2012.10.028.

[ref3] Liu Y. Raman, Mid-IR, and NIR spectroscopic study of calcium sulfates and mapping gypsum abundances in Columbus crater, Mars. Planet. Space Sci. 2018, 163, 35–41. 10.1016/j.pss.2018.04.010.

[ref4] Vaniman D. T.; Martínez G. M.; Rampe E. B.; Bristow T. F.; Blake D. F.; Yen A. S.; Ming D. W.; Rapin W.; Meslin P.-Y.; Morookian J. M.; Downs R. T.; Chipera S. J.; Morris R. V.; Morrison S. M.; Treiman A. H.; Achilles C. N.; Robertson K.; Grotzinger J. P.; Hazen R. M.; Wiens R. C.; Sumner D. Y. Gypsum, bassanite, and anhydrite at Gale crater. Mars 2018, 103 (7), 1011–1020. 10.2138/am-2018-6346.

[ref5] Bishop J. L.; Lane M. D.; Dyar M. D.; King S. J.; Brown A. J.; Swayze G. A. Spectral properties of Ca-sulfates: gypsum, bassanite, and anhydrite. Am. Mineral. 2014, 99 (10), 2105–2115. 10.2138/am-2014-4756.

[ref6] Tang Y.; Gao J.; Liu C.; Chen X.; Zhao Y. Dehydration pathways of gypsum and the rehydration mechanism of soluble anhydrite γ-CaSO_4_. ACS Omega 2019, 4 (4), 7636–7642. 10.1021/acsomega.8b03476. 31459855 PMC6649257

[ref7] Jacques S. D. M.; González-Saborido A.; Leynaud O.; Bensted J.; Tyrer M.; Greaves R. I. W.; Barnes P. Structural evolution during the dehydration of gypsum materials. Mineral. Mag. 2009, 73 (3), 421–432. 10.1180/minmag.2009.073.3.421.

[ref8] Seufert S.; Hesse C.; Goetz-Neunhoeffer F.; Neubauer J. Discrimination of bassanite and anhydrite III dehydrated from gypsum at different temperatures. In Eleventh European Powder Diffraction Conference; Oldenbourg Wissenschaftsverlag: München, 2009, pp 447–452.

[ref9] Seufert S.; Hesse C.; Goetz-Neunhoeffer F.; Neubauer J. Quantitative determination of anhydrite III from dehydrated gypsum by XRD. Cem. Concr. Res. 2009, 39 (10), 936–941. 10.1016/j.cemconres.2009.06.018.

[ref10] López-Buendía A. M.; García-Baños B.; Urquiola M. M.; Catalá-Civera J. M.; Peñaranda-Foix F. L. Evidence of a new phase in gypsum–anhydrite transformations under microwave heating by in situ dielectric analysis and Raman spectroscopy. Phys. Chem. Chem. Phys. 2020, 22 (47), 27713–27723. 10.1039/D0CP04926C. 33242036

[ref11] Shi E.; Wang A.; Li H.; Ogliore R.; Ling Z. Gamma-CaSO_4_ with abnormally high stability from a hyperarid region on Earth and from Mars. J. Geophys. Res.:Planets 2022, 127 (3), e2021JE007108 10.1029/2021je007108.

[ref12] Prieto-Taboada N.; Gómez-Laserna O.; Martínez-Arkarazo I.; Olazabal M. Á.; Madariaga J. M. Raman spectra of the different phases in the CaSO_4_–H_2_O system. Anal. Chem. 2014, 86 (20), 10131–10137. 10.1021/ac501932f. 25226433

[ref13] Bishop J. L.; Yeşilbaş M.; Hinman N. W.; Burton Z. F. M.; Englert P. A. J.; Toner J. D.; McEwen A. S.; Gulick V. C.; Gibson E. K.; Koeberl C. Martian subsurface cryosalt expansion and collapse as trigger for landslides. Sci. Adv. 2021, 7 (6), eabe4459 10.1126/sciadv.abe4459. 33536216 PMC7857681

[ref14] Langevin Y.; Poulet F.; Bibring J.-P.; Schmitt B.; Douté S.; Gondet B. Summer evolution of the north polar cap of Mars as observed by OMEGA/Mars express. Science 2005, 307 (5715), 1581–1584. 10.1126/science.1109438. 15718426

[ref15] Murchie S. L.; Bibring J.-P.; Arvidson R. E.; Bishop J. L.; Carter J.; Ehlmann B. L.; Langevin Y.; Mustard J. F.; Poulet F.; Riu L.; Seelos K. D.; Viviano C. E. Visible to short-wave infrared spectral analyses of Mars from orbit using CRISM and OMEGA. In Remote Compositional Analysis: Techniques for Understanding Spectroscopy, Mineralogy, and Geochemistry of Planetary Surfaces; Bell J. F. III, Bishop J. L., Moersch J. E., Eds.; Cambridge University Press: Cambridge, 2019; pp 453–483.

[ref16] Flahaut J.; Carter J.; Poulet F.; Bibring J. P.; van Westrenen W.; Davies G. R.; Murchie S. L. Embedded clays and sulfates in Meridiani Planum, Mars. Icarus 2015, 248, 269–288. 10.1016/j.icarus.2014.10.046.

[ref17] Christensen P. R.; Wyatt M. B.; Glotch T. D.; Rogers A. D.; Anwar S.; Arvidson R. E.; Bandfield J. L.; Blaney D. L.; Budney C.; Calvin W. M.; Fallacaro A.; Fergason R. L.; Gorelick N.; Graff T. G.; Hamilton V. E.; Hayes A. G.; Johnson J. R.; Knudson A. T.; McSween H. Y.; Mehall G. L.; Mehall L. K.; Moersch J. E.; Morris R. V.; Smith M. D.; Squyres S. W.; Ruff S. W.; Wolff M. J. Mineralogy at Meridiani Planum from the mini-TES experiment on the opportunity rover. Science 2004, 306 (5702), 1733–1739. 10.1126/science.1104909. 15576609

[ref18] Bristow T. F.; Rampe E. B.; Achilles C. N.; Blake D. F.; Chipera S. J.; Craig P.; Crisp J. A.; Des Marais D. J.; Downs R. T.; Gellert R.; Grotzinger J. P.; Gupta S.; Hazen R. M.; Horgan B.; Hogancamp J. V.; Mangold N.; Mahaffy P. R.; McAdam A. C.; Ming D. W.; Morookian J. M.; Morris R. V.; Morrison S. M.; Treiman A. H.; Vaniman D. T.; Vasavada A. R.; Yen A. S. Clay mineral diversity and abundance in sedimentary rocks of Gale crater, Mars. Sci. Adv. 2018, 4 (6), eaar3330 10.1126/sciadv.aar3330. 29881776 PMC5990309

[ref19] Grotzinger J. P.; Sumner D. Y.; Kah L. C.; Stack K.; Gupta S.; Edgar L.; Rubin D.; Lewis K.; Schieber J.; Mangold N.; Milliken R.; Conrad P. G.; DesMarais D.; Farmer J.; Siebach K.; Calef F. 3rd; Hurowitz J.; McLennan S. M.; Ming D.; Vaniman D.; Crisp J.; Vasavada A.; Edgett K. S.; Malin M.; Blake D.; Gellert R.; Mahaffy P.; Wiens R. C.; Maurice S.; Grant J. A.; Wilson S.; Anderson R. C.; Beegle L.; Arvidson R.; Hallet B.; Sletten R. S.; Rice M.; Bell J. 3rd; Griffes J.; Ehlmann B.; Anderson R. B.; Bristow T. F.; Dietrich W. E.; Dromart G.; Eigenbrode J.; Fraeman A.; Hardgrove C.; Herkenhoff K.; Jandura L.; Kocurek G.; Lee S.; Leshin L. A.; Leveille R.; Limonadi D.; Maki J.; McCloskey S.; Meyer M.; Minitti M.; Newsom H.; Oehler D.; Okon A.; Palucis M.; Parker T.; Rowland S.; Schmidt M.; Squyres S.; Steele A.; Stolper E.; Summons R.; Treiman A.; Williams R.; Yingst A. A habitable fluvio-lacustrine environment at Yellowknife Bay, Gale crater, Mars. Science 2014, 343 (6169), 1242777 10.1126/science.1242777. 24324272

[ref20] Wray J. J.; Squyres S. W.; Roach L. H.; Bishop J. L.; Mustard J. F.; Noe Dobrea E. Z. Identification of the Ca-sulfate bassanite in Mawrth Vallis, Mars. Icarus 2010, 209 (2), 416–421. 10.1016/j.icarus.2010.06.001.

[ref21] Harrison T. N. Experimental VNIR reflectance spectroscopy of gypsum dehydration: investigating the gypsum to bassanite transition. Am. Mineral. 2012, 97 (4), 598–609. 10.2138/am.2012.3667.

[ref22] Prasad P. S. R.; Pradhan A.; Gowd T. N. In situ micro-Raman investigation of dehydration mechanism in natural gypsum. Curr. Sci. 2001, 80 (9), 1203–1207.

[ref23] Prasad P. S. R.; Chaitanya V. K.; Prasad K. S.; Rao D. N. Direct formation of the γ-CaSO_4_ phase in dehydration process of gypsum: in situ FTIR study. Am. Mineral. 2005, 90 (4), 672–678. 10.2138/am.2005.1742.

[ref24] Van Driessche A. E. S.; Benning L. G.; Rodriguez-Blanco J. D.; Ossorio M.; Bots P.; García-Ruiz J. M. The role and implications of bassanite as a stable precursor phase to gypsum precipitation. Science 2012, 336 (6077), 69–72. 10.1126/science.1215648. 22491851

[ref25] Worku T.; Parker A. Occurrence of bassanite in lower Lias rocks of the Lyme Regis area, England. Mineral. Mag. 1992, 56 (383), 258–260. 10.1180/minmag.1992.056.383.15.

[ref26] Rapin W.; Meslin P. Y.; Maurice S.; Vaniman D.; Nachon M.; Mangold N.; Schröder S.; Gasnault O.; Forni O.; Wiens R. C.; Martínez G. M.; Cousin A.; Sautter V.; Lasue J.; Rampe E. B.; Archer D. Hydration state of calcium sulfates in Gale crater, Mars: identification of bassanite veins. Earth Planet. Sci. Lett. 2016, 452, 197–205. 10.1016/j.epsl.2016.07.045.

[ref27] Wiens R. C.; Maurice S.; Robinson S. H.; Nelson A. E.; Cais P.; Bernardi P.; Newell R. T.; Clegg S.; Sharma S. K.; Storms S.; Deming J.; Beckman D.; Ollila A. M.; Gasnault O.; Anderson R. B.; André Y.; Michael Angel S.; Arana G.; Auden E.; Beck P.; Becker J.; Benzerara K.; Bernard S.; Beyssac O.; Borges L.; Bousquet B.; Boyd K.; Caffrey M.; Carlson J.; Castro K.; Celis J.; Chide B.; Clark K.; Cloutis E.; Cordoba E. C.; Cousin A.; Dale M.; Deflores L.; Delapp D.; Deleuze M.; Dirmyer M.; Donny C.; Dromart G.; George Duran M.; Egan M.; Ervin J.; Fabre C.; Fau A.; Fischer W.; Forni O.; Fouchet T.; Fresquez R.; Frydenvang J.; Gasway D.; Gontijo I.; Grotzinger J.; Jacob X.; Jacquinod S.; Johnson J. R.; Klisiewicz R. A.; Lake J.; Lanza N.; Laserna J.; Lasue J.; Le Mouélic S.; Legett C.; Leveille R.; Lewin E.; Lopez-Reyes G.; Lorenz R.; Lorigny E.; Love S. P.; Lucero B.; Madariaga J. M.; Madsen M.; Madsen S.; Mangold N.; Manrique J. A.; Martinez J. P.; Martinez-Frias J.; McCabe K. P.; McConnochie T. H.; McGlown J. M.; McLennan S. M.; Melikechi N.; Meslin P.-Y.; Michel J. M.; Mimoun D.; Misra A.; Montagnac G.; Montmessin F.; Mousset V.; Murdoch N.; Newsom H.; Ott L. A.; Ousnamer Z. R.; Pares L.; Parot Y.; Pawluczyk R.; Glen Peterson C.; Pilleri P.; Pinet P.; Pont G.; Poulet F.; Provost C.; Quertier B.; Quinn H.; Rapin W.; Reess J.-M.; Regan A. H.; Reyes-Newell A. L.; Romano P. J.; Royer C.; Rull F.; Sandoval B.; Sarrao J. H.; Sautter V.; Schoppers M. J.; Schröder S.; Seitz D.; Shepherd T.; Sobron P.; Dubois B.; Sridhar V.; Toplis M. J.; Torre-Fdez I.; Trettel I. A.; Underwood M.; Valdez A.; Valdez J.; Venhaus D.; Willis P. The SuperCam instrument suite on the NASA Mars 2020 rover: body unit and combined system tests. Space Sci. Rev. 2020, 217 (1), 4 10.1007/s11214-020-00777-5. 33380752 PMC7752893

[ref28] Murchie S.; Arvidson R.; Bedini P.; Beisser K.; Bibring J. P.; Bishop J.; Boldt J.; Cavender P.; Choo T.; Clancy R. T.; Darlington E. H.; Des Marais D.; Espiritu R.; Fort D.; Green R.; Guinness E.; Hayes J.; Hash C.; Heffernan K.; Hemmler J.; Heyler G.; Humm D.; Hutcheson J.; Izenberg N.; Lee R.; Lees J.; Lohr D.; Malaret E.; Martin T.; McGovern J. A.; McGuire P.; Morris R.; Mustard J.; Pelkey S.; Rhodes E.; Robinson M.; Roush T.; Schaefer E.; Seagrave G.; Seelos F.; Silverglate P.; Slavney S.; Smith M.; Shyong W. J.; Strohbehn K.; Taylor H.; Thompson P.; Tossman B.; Wirzburger M.; Wolff M. Compact reconnaissance imaging spectrometer for Mars (CRISM) on Mars reconnaissance orbiter (MRO). J. Geophys. Res.:Planets 2007, 112, E05S03 10.1029/2006je002682.

[ref29] Bibring J.-P.; Langevin Y.; Gendrin A.; Gondet B.; Poulet F.; Berthé M.; Soufflot A.; Arvidson R.; Mangold N.; Mustard J.; Drossart P. Mars surface diversity as revealed by the OMEGA/Mars express observations. Science 2005, 307 (5715), 1576 10.1126/science.1108806. 15718430

[ref30] Langevin Y.; Poulet F.; Bibring J.-P.; Gondet B. Sulfates in the north polar region of mars detected by OMEGA/Mars express. Science 2005, 307 (5715), 1584–1586. 10.1126/science.1109091. 15718428

[ref31] Massé M.; Bourgeois O.; Le Mouélic S.; Verpoorter C.; Spiga A.; Le Deit L. Wide distribution and glacial origin of polar gypsum on Mars. Earth Planet. Sci. Lett. 2012, 317–318, 44–55. 10.1016/j.epsl.2011.11.035.

[ref32] Weitz C. M.; Bishop J. L.; Grant J. A. Gypsum, opal, and fluvial channels within a trough of Noctis Labyrinthus, Mars: implications for aqueous activity during the Late Hesperian to Amazonian. Planet. Space Sci. 2013, 87, 130–145. 10.1016/j.pss.2013.08.007.

[ref33] Parente M.; Bishop J. L.; Saranathan A. M.; Szynkiewicz A.; Fenton L. K. Detection of bassanite in the North Polar dunes of Mars and implications for aqueous activity. In 53rd Lunar Planet. Sci. Conf., 2022. #2342.

[ref34] Arvidson R. E.; Bibring J.-P.; Bishop J. L.; Carter J.; Ehlmann B. L.; Langevin Y.; Murchie S. L.; Mustard J. F.; Poulet F.; Riu L.; Seelos K. D.; Viviano C. E. Visible to short-wave infrared spectral analyses of Mars from orbit using CRISM and OMEGA. In Remote Compositional Analysis: Techniques for Understanding Spectroscopy, Mineralogy, and Geochemistry of Planetary Surfaces; Bell J. F. III, Bishop J. L., Moersch J. E., Eds.; Cambridge University Press: Cambridge, 2019; pp 453–483.

[ref35] Bishop J. L.; Parente M.; Weitz C. M.; Noe Dobrea E. Z.; Roach L. H.; Murchie S. L.; McGuire P. C.; McKeown N. K.; Rossi C. M.; Brown A. J.; Calvin W. M.; Milliken R.; Mustard J. F. Mineralogy of Juventae Chasma: sulfates in the light-toned mounds, mafic minerals in the bedrock, and hydrated silica and hydroxylated ferric sulfate on the plateau. J. Geophys. Res.:Planets 2009, 114, E00D09 10.1029/2009je003352.

[ref36] Lane M. D.; Bishop J. L.; Dyar M. D.; Hiroi T.; Mertzman S. A.; Bish D. L.; King P. L.; Rogers A. D. Mid-infrared emission spectroscopy and visible/near-infrared reflectance spectroscopy of Fe-sulfate minerals. Am. Mineral. 2015, 100 (1), 66–82. 10.2138/am-2015-4762.

[ref37] De Sanctis M. C.; Altieri F.; Ammannito E.; Biondi D.; De Angelis S.; Meini M.; Mondello G.; Novi S.; Paolinetti R.; Soldani M.; Mugnuolo R.; Pirrotta S.; Vago J. L.; Ma_MISS on ExoMars: mineralogical characterization of the Martian subsurface. Astrobiology 2017, 17 (6–7), 612–620. 10.1089/ast.2016.1541.

[ref38] Bibring J.-P.; Hamm V.; Pilorget C.; Vago J. L.; The MicrOmega investigation onboard ExoMars. Astrobiology 2017, 17 (6–7), 621–626. 10.1089/ast.2016.1642.

[ref39] Rull F.; Maurice S.; Hutchinson I.; Moral A.; Perez C.; Diaz C.; Colombo M.; Belenguer T.; Lopez-Reyes G.; Sansano A.; Forni O.; Parot Y.; Striebig N.; Woodward S.; Howe C.; Tarcea N.; Rodriguez P.; Seoane L.; Santiago A.; Rodriguez-Prieto J. A.; Medina J.; Gallego P.; Canchal R.; Santamaría P.; Ramos G.; Vago J. L.; The Raman laser spectrometer for the ExoMars rover mission to Mars. Astrobiology 2017, 17 (6–7), 627–654. 10.1089/ast.2016.1567.

[ref40] Kosztolanyi C.; Mullis J.; Weidmann M. Measurements of the phase transformation temperature of gypsum-anhydrite, included in quartz, by microthermometry and Raman microprobe techniques. Chem. Geol. 1987, 61 (1), 19–28. 10.1016/0009-2541(87)90022-2.

[ref41] Yeşilbaş M.; Vu T. H.; Hodyss R.; Choukroun M.; Johnson P. V.; Bishop J. L. Characterization of gypsum using vibrational spectroscopy and XRD from low to high temperature and applications to Mars. In 53rd Lunar and Planetary Science Conference (LPSC), 2022. #2396.

[ref42] Fauré N.; Chen J.; Artiglia L.; Ammann M.; Bartels-Rausch T.; Li J.; Liu W.; Wang S.; Kanji Z. A.; Pettersson J. B. C.; Gladich I.; Thomson E. S.; Kong X. Unexpected behavior of chloride and sulfate ions upon surface solvation of Martian salt analogue. ACS Earth Space Chem. 2023, 7 (2), 350–359. 10.1021/acsearthspacechem.2c00204.

[ref43] Marshall W. L.; Slusher R. Thermodynamics of calcium sulfate dihydrate in aqueous sodium chloride solutions, 0–110^°1,2^. J. Phys. Chem. 1966, 70 (12), 4015–4027. 10.1021/j100884a044.

[ref44] Toner J. D.; Catling D. C. A low-temperature aqueous thermodynamic model for the Na–K–Ca–Mg–Cl–SO_4_ system incorporating new experimental heat capacities in Na_2_SO_4_, K_2_SO_4_, and MgSO_4_ solutions. J. Chem. Eng. Data 2017, 62 (10), 3151–3168. 10.1021/acs.jced.7b00265.

[ref45] Ostroff A. G. Conversion of gypsum to anhydrite in aqueous salt solutions. Geochim. Cosmochim. Acta 1964, 28 (9), 1363–1372. 10.1016/0016-7037(64)90154-1.

[ref46] Blounot C. W.; Dickson F. W. The solubility of anhydrite (CaSO_4_) in NaCl-H_2_O from 100 to 450 °C and 1 to 1000 bar. Geochim. Cosmochim. Acta 1969, 33 (2), 227–245. 10.1016/0016-7037(69)90140-9.

[ref47] Brissaud O.; Schmitt B.; Bonnefoy N.; Douté S.; Rabou P.; Grundy W.; Fily M. Spectrogonio radiometer for the study of the bidirectional reflectance and polarization functions of planetary surfaces. 1. Design and tests. Appl. Opt. 2004, 43 (9), 1926–1937. 10.1364/AO.43.001926. 15065723

[ref48] Potin S.; Brissaud O.; Beck P.; Schmitt B.; Magnard Y.; Correia J.-J.; Rabou P.; Jocou L. SHADOWS: a spectro-gonio radiometer for bidirectional reflectance studies of dark meteorites and terrestrial analogs: design, calibrations, and performances on challenging surfaces. Appl. Opt. 2018, 57 (28), 8279–8296. 10.1364/AO.57.008279. 30461780

[ref49] Cloutis E. A.; Hawthorne F. C.; Mertzman S. A.; Krenn K.; Craig M. A.; Marcino D.; Methot M.; Strong J.; Mustard J. F.; Blaney D. L.; Bell J. F. III; Vilas F. Detection and discrimination of sulfate minerals using reflectance spectroscopy. Icarus 2006, 184 (1), 121–157. 10.1016/j.icarus.2006.04.003.

[ref50] Yeşilbaş M.; Boily J.-F. Thin ice films at mineral surfaces. J. Phys. Chem. Lett. 2016, 7 (14), 2849–2855. 10.1021/acs.jpclett.6b01037. 27377606

[ref51] Yeşilbaş M.; Bishop J. L. A molecular perspective for transient liquid salty brine formation in sediments from the McMurdo Dry Valleys, Antarctica, and Applications to Mars. In 52nd Lunar and Planetary Science Conference (LPSC), 2021. #1476.

[ref52] Yeşilbaş M.; Lee C. C.; Boily J.-F. Ice and cryosalt formation in saline microporous clay gels. ACS Earth Space Chem. 2018, 2 (4), 314–319. 10.1021/acsearthspacechem.7b00134.

[ref53] Yeşilbaş M.; Bishop J. L. A molecular view of near surface brines on Mars through mid-infrared spectra of Martian analogs mixed with Cl salts. In 51st Lunar and Planetary Science Conference (LPSC), 2020. #2788.

[ref54] Dalton J. B.; Prieto-Ballesteros O.; Kargel J. S.; Jamieson C. S.; Jolivet J.; Quinn R. Spectral comparison of heavily hydrated salts with disrupted terrains on Europa. Icarus 2005, 177 (2), 472–490. 10.1016/j.icarus.2005.02.023.

[ref55] Ben Mabrouk K.; Kauffmann T. H.; Aroui H.; Fontana M. D. Raman study of cation effect on sulfate vibration modes in solid state and in aqueous solutions. J. Raman Spectrosc. 2013, 44 (11), 1603–1608. 10.1002/jrs.4374.

[ref56] Wang Y.-W.; Kim Y.-Y.; Christenson H. K.; Meldrum F. C. A new precipitation pathway for calcium sulfate dihydrate (gypsum) via amorphous and hemihydrate intermediates. Chem. Commun. 2012, 48 (4), 504–506. 10.1039/C1CC14210K. 21971526

[ref57] Van Driessche A. E. S.; Stawski T. M.; Benning L. G.; Kellermeier M. Calcium sulfate precipitation throughout its phase diagram. In New Perspectives on Mineral Nucleation and Growth: From Solution Precursors to Solid Materials; Van Driessche A. E. S., Kellermeier M., Benning L. G., Gebauer D., Eds.; Springer International Publishing: Cham, 2017; pp 227–256.

[ref58] Jones F. Infrared investigation of Barite and gypsum crystallization: evidence for an amorphous to crystalline transition. CrystEngComm 2012, 14 (24), 8374–8381. 10.1039/c2ce25918d.

[ref59] Vu T. H.; Choukroun M.; Hodyss R.; Johnson P. V. Probing Europa’s subsurface ocean composition from surface salt minerals using in-situ techniques. Icarus 2020, 349, 113746 10.1016/j.icarus.2020.113746.

[ref60] Malkin T. L.; Murray B. J.; Brukhno A. V.; Anwar J.; Salzmann C. G. Structure of ice crystallized from supercooled water. Proc. Natl. Acad. Sci. U.S.A. 2012, 109 (4), 1041–1045. 10.1073/pnas.1113059109. 22232652 PMC3268266

[ref61] Ritterbach L.; Becker P. Temperature and humidity dependent formation of CaSO_4_·*x*H_2_O (*x* = 0, ..., 2) phases. Global Planet. Change 2020, 187, 103132 10.1016/j.gloplacha.2020.103132.

[ref62] Oetzel M.; Scherberich F.-D.; Heger G. Heating device for high temperature X-ray powder diffraction studies under controlled water vapour pressure (0–1000 mbar) and gas temperature (20–200 °C). Powder Diffr. 2000, 15 (1), 30–37. 10.1017/S0885715600010800.

[ref63] Yeşilbaş M.; Bishop J. L. The spectral properties of gypsum from −90 to 400 °C and implications for Mars. In 52nd Lunar and Planetary Science Conference (LPSC), 2021. #1968.

[ref64] Strydom C. A.; Hudson-Lamb D. L.; Potgieter J. H.; Dagg E. The thermal dehydration of synthetic gypsum. Thermochim. Acta 1995, 269–270, 631–638. 10.1016/0040-6031(95)02521-9.

[ref65] Kyono A.; Ikeda R.; Takagi S.; Nishiyasu W. Structural evolution of gypsum (CaSO_4_·2H_2_O) during thermal dehydration. J. Mineral. Petrol. Sci. 2022, 117 (1), 220811 10.2465/jmps.220811.

[ref66] McMahon S.; Parnell J.; Reekie P. B. R. Mars-analog calcium sulfate veins record evidence of ancient subsurface life. Astrobiology 2020, 20 (10), 1212–1223. 10.1089/ast.2019.2172. 32985907

[ref67] Bishop J. L.; Fairén A. G.; Michalski J. R.; Gago-Duport L.; Baker L. L.; Velbel M. A.; Gross C.; Rampe E. B. Surface clay formation during short-term warmer and wetter conditions on a largely cold ancient Mars. Nat. Astron. 2018, 2 (3), 206–213. 10.1038/s41550-017-0377-9. PMC7008931 32042926

[ref68] Kounaves S. P.; Hecht M. H.; Kapit J.; Quinn R. C.; Catling D. C.; Clark B. C.; Ming D. W.; Gospodinova K.; Hredzak P.; McElhoney K.; Shusterman J. Soluble sulfate in the martian soil at the Phoenix landing site. Geophys. Res. Lett. 2010, 37, L09201 10.1029/2010gl042613.

[ref69] Rapin W.; Ehlmann B. L.; Dromart G.; Schieber J.; Thomas N. H.; Fischer W. W.; Fox V. K.; Stein N. T.; Nachon M.; Clark B. C.; Kah L. C.; Thompson L.; Meyer H. A.; Gabriel T. S. J.; Hardgrove C.; Mangold N.; Rivera-Hernandez F.; Wiens R. C.; Vasavada A. R. An interval of high salinity in ancient Gale crater lake on Mars. Nat. Geosci. 2019, 12 (11), 889–895. 10.1038/s41561-019-0458-8.

[ref70] Reiss A. G.; Gavrieli I.; Rosenberg Y. O.; Reznik I. J.; Luttge A.; Emmanuel S.; Ganor J. Gypsum precipitation under saline conditions: thermodynamics, kinetics, morphology, and size distribution. Minerals 2021, 11 (2), 141 10.3390/min11020141.

