# Supplementary material for: Geochemical Transformations of Gypsum Under Multiple Environmental Settings and Implications for Ca-Sulfate Detection on Mars
Source: ACS Earth Space Chem. 2025 Feb 28;9(3):433–44. doi: 10.1021/acsearthspacechem.4c00137 (PMC11931525; doi:10.1021/acsearthspacechem.4c00137)
Supplement: Supplementary file 1 — sp4c00137_si_001.pdf [file sp4c00137_si_001.pdf]

Begränsad delning

Supplementary Information

Geochemical Transformations of Gypsum under Multiple Environmental Settings and Implications for Ca-Sulfate Detection on Mars

Merve Yeşilbaş[1,2*], Tuan H. Vu[3], Robert Hodyss[3], Olivier Poch[4], Bernard Schmitt[4], Mathieu Choukroun[3], Paul V. Johnson[3] and Janice L. Bishop[1,5]

[1] Carl Sagan Center, SETI Institute, Mountain View, CA 94043, USA.
[2*] Department of Chemistry, Umeå University, Umeå, SE 90187, Sweden.
[3] Jet Propulsion Laboratory, California Institute of Technology, 4800 Oak Grove Drive, Pasadena, CA 91109, USA.
[4] University of Grenoble Alpes, CNRS, IPAG, 38000, Grenoble, France.
[5] NASA Ames Research Center, Moffett Field, CA 94035, USA.

*Corresponding author: merve.yesilbas@umu.se

tel. +46-722195760.

Characterization of gypsum, Raman, XRD, SEM and others.

Submitted to the ACS Earth and Space Chemistry

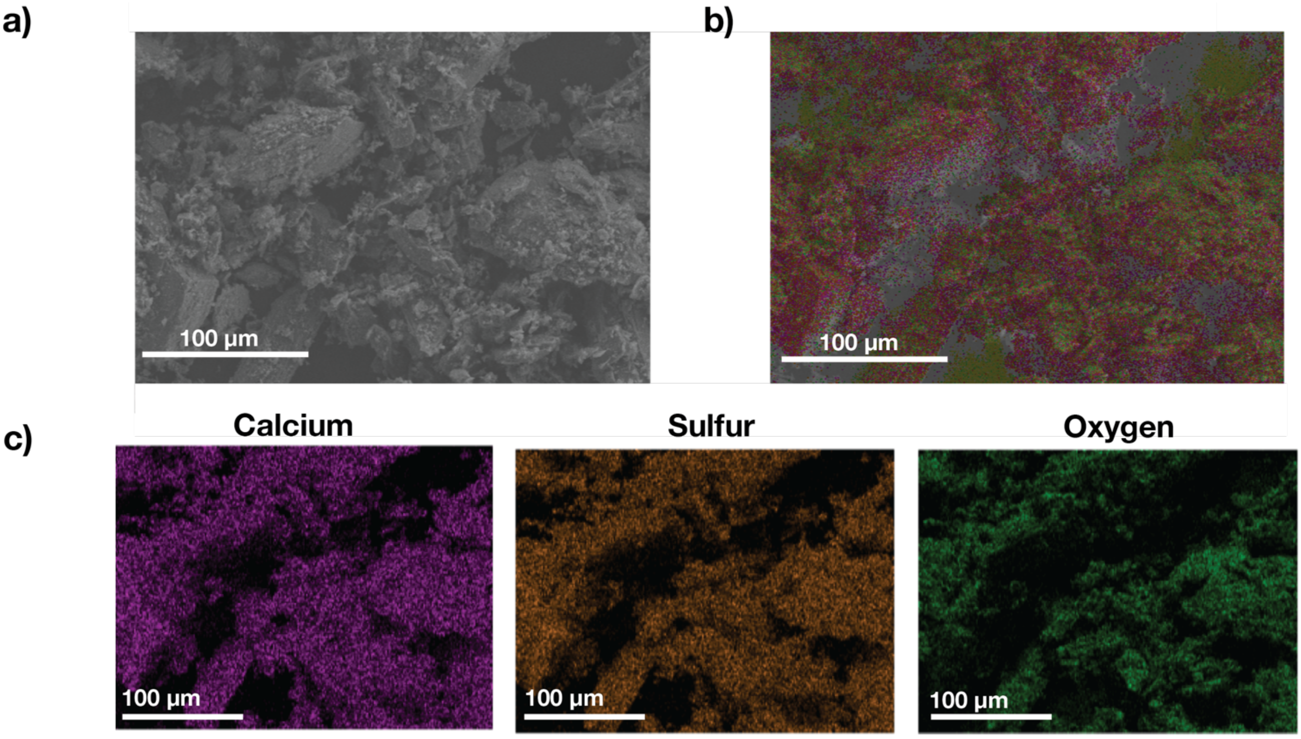

Figure S1. a) SEM image of gypsum (JB1464-f) used in this study. **b)** Elemental mapping and distribution of chemicals in gypsum collected by SEM- Energy Dispersive Spectroscopy (EDS). **c)** Chemical distribution of calcium, sulfur and oxygen elements in gypsum.

Image Credit: Merve Yesilbas (Umeå University). We thank the assistance and help from the engineers at Umeå Core Facility for Electron Microscopy (UCEM), Umeå University.

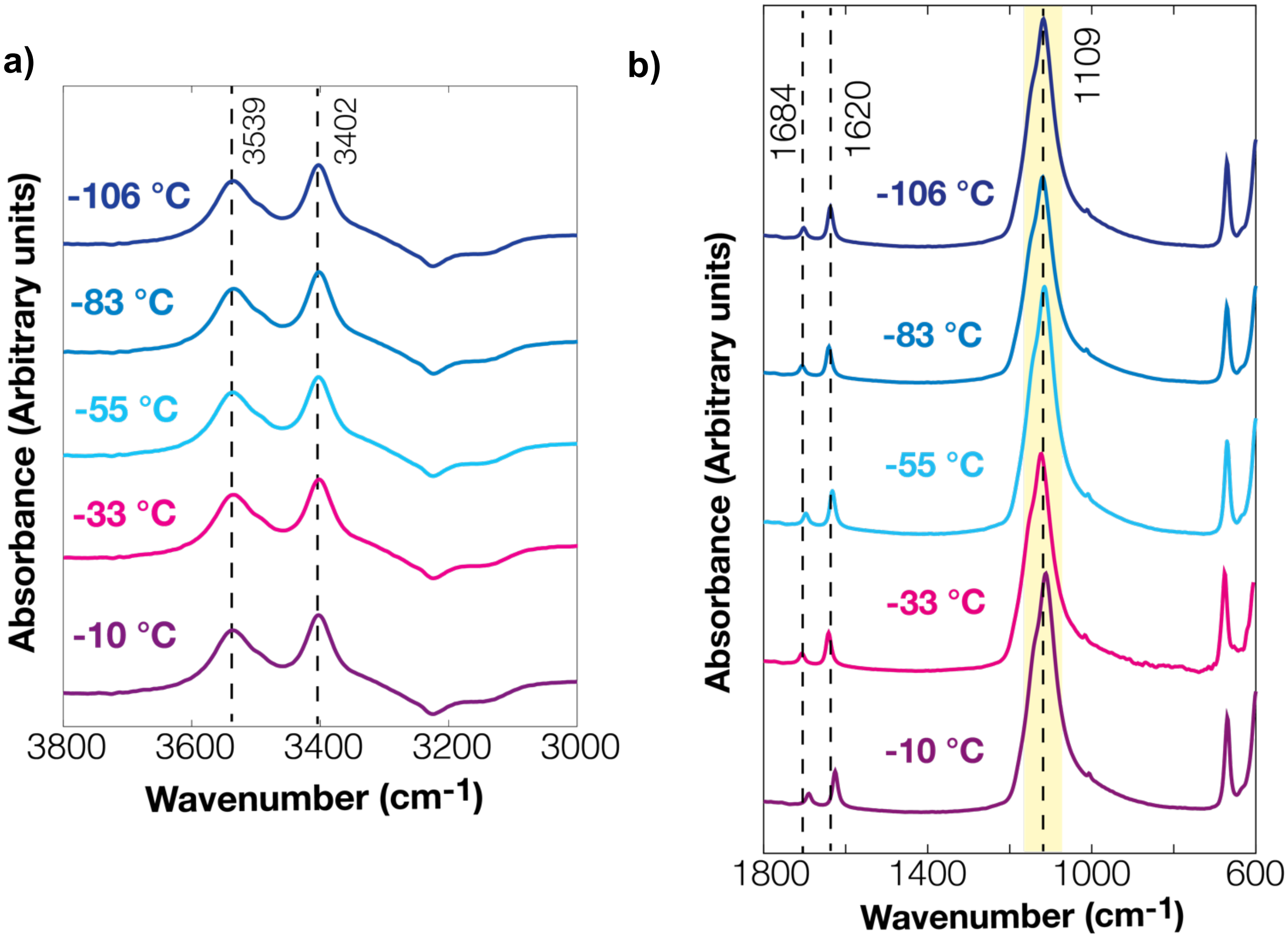

Figure S2. Cryogenic ATR-FTIR spectra of flash-frozen gypsum (JB1464-f) paste from 25 to -106 °C used in this study. **a)** The IR spectra from H_2O stretching region at given temperatures. **b)** The IR spectra from the H_2O bending and SO_4 stretching region at given temperatures. Experiments were performed by Merve Yesilbas in the Umeå University.

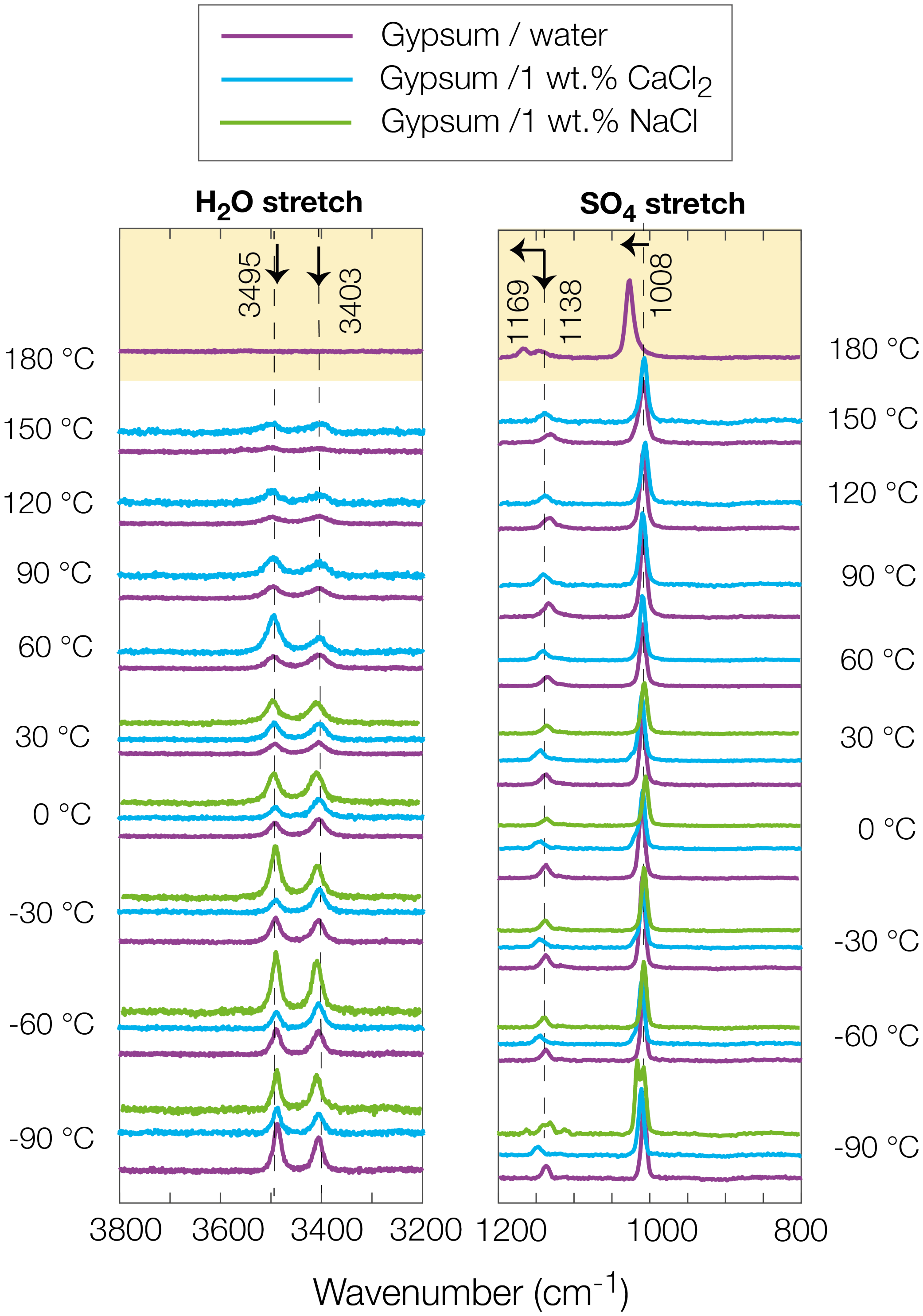

Figure S3. Cryogenic Raman spectra collected from -90 to upon 180 °C for gypsum paste, -90 to 30 °C for gypsum-NaCl paste and -90 to 150 °C for gypsum-$CaCl_2$ paste. Experiments were performed by Merve Yesilbas in the facilities at the Jet Propulsion Laboratory.

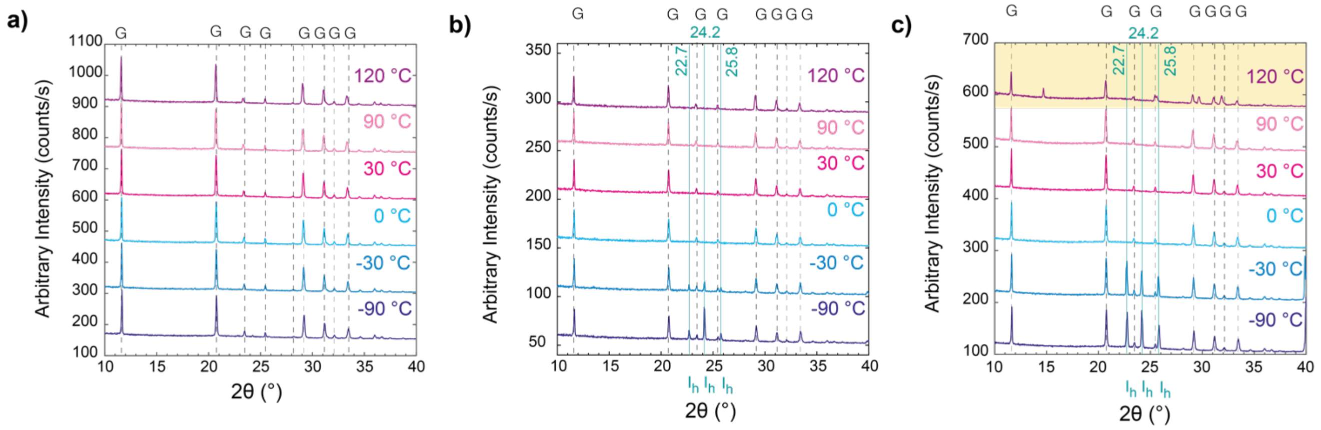

Figure S4. a) Cryogenic XRD patterns of gypsum-water paste; **b)** gypsum-NaCl paste; **c)** gypsum-$CaCl_2$ paste heated from -90 to 120 °C with applying 6 °C/min heating rate. Experiments were performed by Tuan H. Vu and Merve Yesilbas at the facilities at Jet Propulsion Laboratory.
